# Aptamer-Based Smart Targeting and Spatial Trigger–Response Drug-Delivery Systems for Anticancer Therapy

**DOI:** 10.3390/biomedicines12010187

**Published:** 2024-01-15

**Authors:** Dongsik Park, Su Jin Lee, Jee-Woong Park

**Affiliations:** 1 Drug Manufacturing Center, Daegu-Gyeongbuk Medical Innovation Foundation (K-MEDI Hub), Daegu 41061, Republic of Korea; 2 Medical Device Development Center, Daegu-Gyeongbuk Medical Innovation Foundation (K-MEDI Hub), Daegu 41061, Republic of Korea

**Keywords:** aptamer, targeted drug delivery, stimuli-responsive drug delivery

## Abstract

In recent years, the field of drug delivery has witnessed remarkable progress, driven by the quest for more effective and precise therapeutic interventions. Among the myriad strategies employed, the integration of aptamers as targeting moieties and stimuli-responsive systems has emerged as a promising avenue, particularly in the context of anticancer therapy. This review explores cutting-edge advancements in targeted drug-delivery systems, focusing on the integration of aptamers and stimuli-responsive platforms for enhanced spatial anticancer therapy. In the aptamer-based drug-delivery systems, we delve into the versatile applications of aptamers, examining their conjugation with gold, silica, and carbon materials. The synergistic interplay between aptamers and these materials is discussed, emphasizing their potential in achieving precise and targeted drug delivery. Additionally, we explore stimuli-responsive drug-delivery systems with an emphasis on spatial anticancer therapy. Tumor microenvironment-responsive nanoparticles are elucidated, and their capacity to exploit the dynamic conditions within cancerous tissues for controlled drug release is detailed. External stimuli-responsive strategies, including ultrasound-mediated, photo-responsive, and magnetic-guided drug-delivery systems, are examined for their role in achieving synergistic anticancer effects. This review integrates diverse approaches in the quest for precision medicine, showcasing the potential of aptamers and stimuli-responsive systems to revolutionize drug-delivery strategies for enhanced anticancer therapy.

## 1. Introduction

Cancer remains one of the most formidable challenges in modern medicine, necessitating innovative approaches for more effective and targeted therapeutic interventions. Over the years, the convergence of nanotechnology, biochemistry, and materials science has paved the way for the development of sophisticated drug-delivery systems designed to overcome the limitations of conventional cancer treatments. In this context, the exploration of smart targeting and spatial trigger–response mechanisms has emerged as a promising frontier, offering the potential to enhance the precision and efficacy of anticancer therapies.

The conventional chemotherapy paradigm often suffers from systemic toxicity, as potent cytotoxic agents are indiscriminately delivered throughout the body, causing adverse effects on healthy tissues. To address this limitation, the integration of smart targeting mechanisms has gained prominence. Within this paradigm, aptamers, short nucleic acid sequences with a unique ability to bind specifically to target molecules, have emerged as valuable targeting ligands. Aptamers share similarities with antibodies as they exhibit a high affinity for specific targets, making them a focus of research in disease-targeted therapy owing to their remarkable selectivity [1,2,3]. Regarded as promising therapeutic agents, aptamers possess attributes such as non-immunogenicity, high specificity, and stability. To mitigate systemic toxicity, aptamers can be directly linked with drugs or integrated into nanocarriers. In addition to being cost-effective, aptamers are easily modifiable and considered as “chemical antibodies.” Aptamers offer a tailored approach to drug delivery by selectively recognizing cancer-specific biomarkers, thereby facilitating the precise delivery of therapeutic payloads to malignant cells.

For precision in anticancer therapy, in addition to smart targeting with aptamers, strategies for spatiotemporal control over drug release have been studied. Conventional cancer therapies, such as chemotherapy and radiation, often exhibit limitations related to non-specificity, resulting in adverse effects on healthy tissues and systemic toxicity. Spatial anticancer therapy, on the other hand, is designed to overcome several limitations by precisely delivering therapeutic agents to specific regions within the body, primarily the tumor site. Spatial trigger–response drug-delivery systems, discussed in this review, aim to overcome the challenges associated with conventional drug delivery by responding to specific cues within the tumor microenvironment (TME) or external stimuli.

## 2. Targeted Drug-Delivery Systems with Aptamers

### 2.1. Aptamers

In 1990, a groundbreaking development marked the emergence of aptamers as synthetic ligands endowed with the capacity for molecular recognition [1,2]. Aptamers, in essence, represent sophisticated analogs of antibodies, consisting of concise single-stranded oligonucleotides capable of selectively and strongly binding to proteins, peptides, metal ions, and cells. This exceptional selectivity and high affinity stem from their distinctive three-dimensional conformation, which is achieved through intramolecular interactions. Furthermore, aptamers are garnering increasing attention in the realm of drug targeting due to their advantages such as low toxicity, minimal or absent immunogenicity, and more economical production, particularly when compared to antibodies [3]. 

Aptamers have been synthesized using the Systematic Evolution of Ligands by Exponential Enrichment (SELEX) technique [4,5]. This method enables the generation of aptamers capable of specifically targeting proteins that are overexpressed on the surface of particular cells, analogous to a donor–receptor system involving cancer cells. The SELEX technique can enhance the aptamers’ ability to bind to known or unidentified receptors located on the cell membrane [6]. The binding affinity of an aptamer is typically characterized by dissociation rate constants (kd), which are associated with dissociation and association rate constants (koff/kon). Moreover, aptamers can be functionalized with a variety of materials on the intended sites. Furthermore, aptamers with small molecular weights can be rapidly eliminated through renal filtration in vivo [7]. Aptamer sequences readily accommodate the loading of antitumor drugs targeted specifically to cancer cells.

Hence, researchers have employed biological, biocompatible, and biodegradable carrier materials like lipids, lactic acid, or chitosan, as well as various approved non-toxic polymers (owing to their ease of production), or synthetic materials such as titanium dioxide (TiO_2_) or cadmium (Cd) as a means to enhance the loading of anticancer agents [8]. 

Zeng et al. formed aptamer–drug conjugates with doxorubicin (DOX) and an aptamer for targeted cancer therapy in vitro and in vivo [9]. Synthesizing tripartite newkome-type monomers (TNMs) of DOX with pH-sensitive hydrazone bonds improves therapeutic potential. Apt–TNM-DOX, created via a self-loading process, stably carries 15 DOX molecules per aptamer. The pH-dependent DOX release at a pH level of 5.0 targets lymphoma cells selectively, minimizing off-target effects. Apt–TNM-DOX, a non-toxic approach, shows promise for aptamer-based targeted therapeutics, potentially reducing chemotherapy’s non-specific side effects.

It is worth highlighting that the use of PEGylated-functionalized materials can reduce systemic clearance and enhance the stability of nanoparticles, which is crucial for the passive targeting of tumors by nanocarriers [10]. Furthermore, the incorporation of different types of aptamer-based modifications on the surface of polymeric nanocarriers can result in heightened selectivity and sensitivity when targeting specific organs, tissues, and other sites [11]. 

### 2.2. Aptamer-Modified Nanomaterials

**Figure 1:**
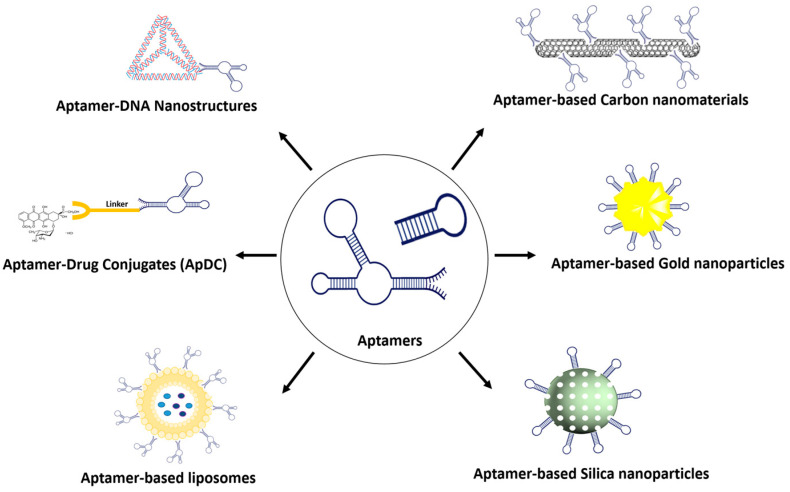
A variety of aptamer conjugates for targeted drug-delivery system.

Because of their distinctive physicochemical attributes, exceedingly small size, expansive surface area, and exceptional loading capacity, nanomaterials surmount numerous constraints associated with conventional therapeutic and diagnostic approaches [12]. The essence of advancing nanomedicine lies in enhancing the specific recognition capability for pathological tissues. The conjugation of aptamers with nanomaterials signifies a noteworthy advancement in targeted drug delivery (Figure 1) [13]. In this section, various representative aptamer-based nanomaterials employed for drug delivery will be discussed. 

#### 2.2.1. Aptamer–Gold Conjugation 

Being a significant nanomaterial, gold nanoparticles have garnered substantial attention in the field of biomedicine owing to their elevated surface-to-volume ratio, low toxicity, outstanding stability, and biological compatibility [14,15]. Aptamer-conjugated gold nanomaterials (Apt-AuNPs), combining the distinctive advantageous features of aptamers and gold nanoparticles, have found extensive application in cancer diagnosis and therapy [16].

Since the investigation by the Mirkin group utilized DNA molecules to construct a polymeric network of nanoparticles [17], numerous studies have emerged, including the development of enzyme-responsive Apt-AuNPs for the detection of mucin 1 protein (MUC1) [18], the utilization of Apt-AuNPs in conjunction with graphene oxide for the photothermal therapy of breast cancer [19], and the design of an aptamer-functionalized AuNPs-Fe_3_O_4_-GS capture probe for monitoring circulating tumor cells in whole blood [20].

In a recent study, Khorshid et al. investigated the utilization of gold nanoparticles functionalized with an anti-HER-2 aptamer for the precise delivery of dasatinib (DSB) to breast cancer cells [21]. The loading efficiency of the activated drug on both plain and porous gold nanoparticles was notably elevated (52% and 68%, respectively) compared to that of free DSB within gold nanoparticles (1 to 2.5%). Porous gold nanoparticles functionalized with the aptamer and loaded with activated dasatinib demonstrated heightened cytotoxicity and enhanced cellular uptake in comparison to nanoparticles containing modified DSB or unactivated DSB.

Specifically targeted gold nanobipyramids (GNBs) demonstrate potential as photothermal therapeutic agents and have diverse applications such as contrast agents, biosensors, and drug-delivery vehicles. Navyatha et al. explored the efficiency of targeting moieties (aptamers and antibodies) in specifically targeting the MUC1 protein and their impact on cytotoxicity [22]. The results indicate that aptamer-conjugated GNBs exhibit reduced cytotoxicity compared to antibody-conjugated ones. Aptamer-conjugated GNBs are more effective in photoablating MCF7 cell lines than HCT116 cell lines, highlighting their potential for targeted photothermal therapy.

#### 2.2.2. Aptamer–Silica Conjugation

Silica nanoparticles have emerged as feasible carriers in drug-delivery systems [23]. These particles have effectively enabled controlled drug release both in vivo and in vitro, achieved through pH and temperature variations, photochemical reactions, and specific redox reactions [24]. Integrated with aptamers, the silica nanoparticles have demonstrated the ability to amplify the therapeutic effects against cancer with a reduced dosage of the drug [25].

Recently, Heydari et al. studied surface-modified mesoporous silica nanoparticles (MSNs) for the targeted delivery of anticancer agents (daunorubicin and cytarabine) to K562 leukemia cancer cells [26]. The MSNs were further enhanced with the KK1B10 aptamer (Apt) to improve uptake by K562 cells through ligand–receptor interactions. MSNs coated with CS and conjugated with the aptamer exhibited a significantly lower IC_50_ value of 2.34 µg/mL compared to MSNs without the aptamer conjugation (IC_50_ value of 12.27 µg/mL). The aptamer-modified MSNs exhibited lower IC_50_ values against cancer cell lines and demonstrated enhanced anticancer activity in animal models, highlighting their potential as effective targeted anticancer agents with controlled drug release properties.

Kianpour et al. studied the oncoprotein cell migration-inducing hyaluronidase 2 (CEMIP2) in colorectal cancer (CRC) and developed an aptamer-based silica nanoparticle for targeted therapy [27]. The cell-SELEX technique identified aptCEMIP2(101), which specifically interacts with full-length CEMIP2. Treatment with aptCEMIP2(101) reduced CEMIP2-mediated tumorigenesis in vitro. Mesoporous silica nanoparticles (MSN) carrying aptCEMIP2(101) and Dox significantly suppressed tumorigenesis, with Dox@MSN-aptCEMIP2(101) showing higher efficacy compared to Dox@MSN and MSN-aptCEMIP2(101) in CRC-derived cells. This study revealed CEMIP2 as a novel oncogene and introduced an effective aptamer-based targeted therapeutic strategy.

Xie et al. focused on enhancing the targeted delivery of doxorubicin (DOX) to colon cancer cells using aptamer-modified mesoporous silica nanoparticles (Ap-MSN-DOX) [28]. The nanoparticles were characterized for various properties, and results demonstrated increased binding to EpCAM-overexpressing SW620 colon cancer cells. This led to enhanced cellular uptake and cytotoxicity compared to non-aptamer-modified nanoparticles (MSN-DOX). Ap-MSN-DOX also exhibited the significant inhibition of EpCAM expression on SW620 cells, indicating its potential for targeted delivery to improve therapeutic efficacy while minimizing side effects.

#### 2.2.3. Aptamer–Carbon Conjugation 

**Figure 2:**
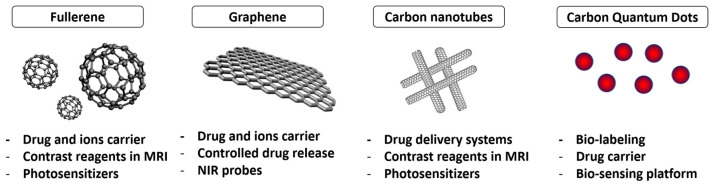
Carbon nanomaterials (CNMs) for theranostics.

Carbon nanomaterials such as graphene and carbon nanotubes are highly valued for their mechanical, optical, and thermal properties for various biomedical applications [29] (Figure 2).

The conjugation of aptamers with carbon nanomaterials (CNMs) has given rise to theranostic agents [30], revolutionizing personalized cancer medicine [31], target-specific imaging, and the label-free diagnosis of diverse cancers [32]. Aptamer-functionalized CNMs serve as nanovesicles for the precise delivery of anticancer agents, such as doxorubicin [33] and 5-fluorouracil [34], directly to tumor sites. This innovative approach holds promise for advanced, targeted therapeutic strategies with reduced side effects in medical treatments.

Zavareh et al. developed a targeted drug-delivery system, the 5-fluorouracil-chitosan-carbon quantum dot-aptamer (5-FU-CS-CQD-Apt) nanoparticle, using a water-in-oil emulsification method for breast cancer treatment [34]. Characterized by high drug-loading and entrapment efficiency, the nanoparticle exhibited an average size of 122.7 nm and a zeta potential of +31.2 mV. In vitro studies demonstrated controlled drug release, and functional assays indicated the biocompatibility of the blank nanoparticle and the efficient tumor cell-killing capabilities of 5-FU-CS-CQD-Apt, making it a potential carbon nanocarrier for breast cancer treatment.

Zhao et al. introduced aptamer-functionalized Fe_3_O_4_@carbon@doxorubicin nanoparticles (Apt-Fe_3_O_4_@C@DOX) for synergistic chemo–photothermal cancer therapy [33]. The nanoparticles exhibit high photothermal conversion efficiency and pH/heat-induced drug release. In vitro experiments demonstrate enhanced toxicity towards lung adenocarcinoma cells (A549) with combined chemo–photothermal therapy compared to individual treatments. Furthermore, the nanoparticles exhibit decreasing contrast enhancement in magnetic resonance imaging, suggesting potential applications as contrast agents for the T2-weighted imaging of tumor tissues. Apt-Fe_3_O_4_@C@DOX nanoparticles hold significant promise for cancer therapy.

Fullerenes composed of 60 and 70 carbon atoms are spherical carbon allotropes commonly utilized in drug-delivery systems like carbon nanotubes (CNTs) and graphene. With a size around 1 nm, their geometry and surface area make them suitable for drug release applications [35]. Fullerenes, featuring a stable ellipsoidal structure with apolar properties, allow for modifications with various drugs or biomolecules, including radioactive atoms for diagnostic purposes. Notably, C60 exhibits a high stability due to the delocalization of π electrons in benzene rings, showing minimal toxicity in in vitro and in vivo cytotoxicity studies.

Fullerene-based photosensitizers, particularly trimalonic acid-modified C70 fullerene (TF70), show significant potential in photodynamic therapy (PDT) [36]. An aptamer-guided TF70 photosensitizer demonstrates enhanced PDT efficiency against A549 lung cancer cells, even in the presence of serum. The conjugation of the aptamer (R13) improves the lysosomal localization of TF70-R13, leading to the increased production of intracellular reactive oxygen species (ROS) under light irradiation, effectively killing cells. TF70-R13’s enhanced photodynamic efficiency and good biocompatibility position it as a highly promising tumor-specific photosensitizer for PDT.

Carbon nanotubes (CNTs) have garnered considerable interest as potential nanocarriers for drug delivery [37]. Their distinctive properties, such as an ultrahigh length-to-diameter ratio and efficient cellular uptake, make them promising nanocarriers in this field. The unique conjugated structure of CNTs is well-suited for π–π stacking interactions with various drugs and therapeutic molecules that possess aromatic rings, including anthracyclines. This interaction enhances the potential for the effective loading and delivery of such drugs, contributing to the attractiveness of CNTs as nanocarriers in drug-delivery applications.

Chen et al. developed an aptamer-siRNA chimera (Chim), polyethyleneimine (PEI), 5-fluorouracil (5-FU), carbon nanotube (CNT), and collagen membrane, which demonstrated sustained 5-FU release for over two weeks [38]. The aptamer-siRNA chimera enabled specific binding to gastric cancer cells, facilitating the targeted delivery of 5-FU and silencing drug-resistant genes. In vitro experiments revealed that Chim/PEI/5-FU/CNT nanoparticles induced apoptosis in 5-FU-resistant gastric cancer cells, inhibiting their invasion and proliferation. Animal studies showed the significant inhibition of mitogen-activated protein kinase (MAPK) expression and effective treatment of peritoneal dissemination of 5-FU-resistant gastric cancer.

Graphene oxides (GOs) have emerged as promising drug carriers for targeted delivery systems due to their good endocytosis, biocompatibility, and ample surface area for drug loading [39]. GO’s dispersing capability in water and physiological environments, attributed to its abundant functional groups like epoxide, hydroxyl, and carboxyl groups, enhances its appeal. The interaction between these groups and drug functional groups, including hydrogen bonding, π−π stackings, and hydrophobic interactions, facilitates efficient drug loading onto the nanocarrier.

Shahidi et al. engineered drug-delivery cargo by decorating carboxylated graphene oxide (cGO) with an aptamer, HB5, for the simultaneous delivery of DOX and silibinin (Sili) in a combination therapy against MCF-7 and SK-BR-3 breast cancer cells [40]. Apt-cGO exhibited a sheet-like nanostructure with a high entrapment efficiency for both Sili (70.42%) and DOX (84.22%). The nanocomposites, selectively taken up by breast cancer cells, released both drugs upon the cleavage of the cGO–drug interaction. Apt-cGO-DOX-Sili nanocomposites demonstrated higher in vitro cytotoxicity than free drugs, suppressing cancer cell survival signals and inducing apoptosis, suggesting a promising drug-delivery approach for breast chemotherapy.

Ganoderenic acid D (GAD) from *Ganoderma lucidum*, which is known to show anticancer activity [41], was loaded onto a graphene oxide-polyethylene glycol-anti-epidermal growth factor receptor (GO-PEG-EGFR) carrier to create a targeting antitumor nanocomposite (GO-PEG@GAD) [42]. The carrier, modified with anti-EGFR aptamer, achieved a high loading content (77.3%) and encapsulation efficiency (89.1%). Targeting to HeLa cells was confirmed in vitro and in vivo. The subcutaneously implanted tumor mass significantly decreased by 27.27% after GO-PEG@GAD treatment. The nanocomposite’s in vivo anti-cervical carcinoma activity was attributed to the activation of the intrinsic mitochondrial pathway.

A pH-sensitive nano-graphene oxide (nGO)-based system was developed for delivering Curcumin (Cur) to MCF cancer cells [43]. Cur is loaded onto nGO, decorated with bovine serum albumin (BSA) for improved stability and protection, and functionalized with the AS1411 aptamer. The system exhibits 8.9% drug loading, 78.9% loading efficiency, and preferential release in acidic conditions. MTT tests show growth inhibition, with the AS1411 aptamer enhancing efficiency toward MCF7 cells due to its significant affinity for highly expressed nucleolin on MCF7 plasma membranes.

### 2.3. Aptamer–Drug Conjugate

Aptamers, known for high specificity, show promise in targeted therapy. They excel in delivering therapeutic agents against toxins or hypoimmunogenic agents, surpassing current antibody techniques. Aptamers serve as carriers for aptamer–drug conjugates (ApDCs), offering advantages in targeted drug delivery [44,45]. Similar to antibodies in recognition, aptamers allow for the design of various ApDCs, typically comprising an aptamer, linker, and drug. Aptamers guide therapeutic delivery to disease sites, modulating target biomarker functions. Their chemical stability, simplicity of modification, and molecular engineering enable versatile conjugation with therapeutics. ApDCs, effectively inhibiting tumor growth in vitro and in vivo, present a compelling avenue for targeted therapy.

In pursuit of an enhanced drug-targeting approach, Gray et al. developed a novel class of targeted anticancer therapeutics—aptamers linked to potent chemotherapeutics [46]. The E3 aptamer, selected for its specificity to prostate cancer cells, was successfully conjugated to monomethyl auristatin E (MMAE) and monomethyl auristatin F (MMAF). This resulting cytotoxic agent effectively killed prostate cancer cells in vitro while sparing normal cells. In vivo, the E3 aptamer targeted tumors, and the MMAF–E3 conjugate significantly curtailed prostate cancer growth in mice. Additionally, antidotes were introduced to counteract unintended cytotoxicity, serving as a safety switch in vivo.

The Sgc8-c aptamer, binding to PTK7, enables the recognition of haemato-oncological malignancies. Aptamer–drug conjugates, specifically Sgc8-c-carb-da, were developed by hybridizing Sgc8-c with dasatinib for lymphoma chemotherapy [47]. This conjugate demonstrated the targeted inhibition of lymphocyte growth, inducing cell death, proliferation arrest, and affecting mitochondrial potential. In an in vitro assay mimicking in vivo conditions, Sgc8-c-carb-da exhibited 2.5-fold higher cytotoxic effects than dasatinib, offering a promising therapeutic concept for lymphoma and highlighting opportunities for novel targeted biotherapeutics through chemical synthesis.

The human transferrin receptor-targeted DNA aptamer (HG1-9)-fluorophore conjugates were utilized for visualizing their internalization and intracellular transport [48]. Unlike transferrin, these aptameric conjugates demonstrated prolonged cellular retention, escaping degradation in late endosomes or lysosomes. About 90% of internalized HG1-9 was retained in cellular vesicles at pH levels between 6.0 and 6.8, facilitating efficient drug release. These results highlight HG1-9 as a versatile tool for the specific and effective delivery of diverse therapeutics with accurate release.

Henri et al. investigated a DNA aptamer targeting the cancer biomarker EpCAM for delivering chemotherapy [49]. Findings suggest EpCAM aptamers effectively bind to epithelial ovarian cancer, providing a tunable ligand alternative with specificity and sensitivity. Aptamers demonstrated cytotoxicity in monolayer, tumorsphere, and tumor-enriching assays, highlighting their potential for cancer therapeutics. The study supports aptamers’ adaptability through post-SELEX engineering and proposes their role in developing targeted drug delivery for novel cancer treatments.

A multivalent nanomedicine, HApt-tFNA@Dxd, was developed by combining the anti-HER2 aptamer (HApt), tetrahedral framework nucleic acid (tFNA), and deruxtecan (Dxd) [50]. HApt-tFNA@Dxd exhibited enhanced structural stability, targeted cytotoxicity to HER2-positive gastric cancer, and improved tissue aggregation in tumors compared to free Dxd and tFNA@Dxd. The study represents a significant advance in developing DNA-based nanomaterials for HER2-positive cancer therapy, showcasing HApt-tFNA@Dxd as a promising chemotherapeutic medicine.

Jo et al. developed a tumor-specific bifunctional G-Quadruplex aptamer(BGA) with a dual function, inhibiting topoisomerase 1 (TOP1) and targeting nucleolin (NCL)-positive MCF-7 cells [51]. The BGA–DM1 conjugate demonstrated a 20-fold stronger anticancer effect than free DM1 and was even 10-fold stronger than AS1411 (NCL aptamer)-DM1. The research suggests that biased libraries can yield aptamers with effector functions for developing potent aptamer–drug conjugates, offering a distinct approach to targeted cancer therapy with synergistic effects compared to traditional antibody–drug conjugates.

Liu et al. introduced a CD71/CD44 dual-aptamer-gemcitabine (CD71-CD44-GEMs) conjugate for treating bladder cancer by co-targeting cancer cells and cancer stem cells (CSCs) [52]. Evaluations demonstrated CD71-CD44-GEMs’ selective binding and significant inhibitory effects on bladder cancer in vitro and in vivo. The conjugate outperformed single-target GEM conjugates (CD71-GEMs or CD44-GEMs) in terms of binding affinity and inhibitory efficacy, making CD71-CD44-GEMs a promising approach for treating bladder cancer by effectively targeting both cancer cells and CSCs.

Lysosome-targeting chimeras (LYTACs) offer a promising avenue for targeted protein degradation, extending to extracellular targets. However, the conventional method involving the antibody-trivalent N-acetylgalactosamine (tri-GalNAc) conjugation is complex and time-consuming. Addressing these challenges, Wu et al. introduced aptamer-based LYTACs (Apt-LYTACs), enabling the efficient and rapid degradation of the extracellular protein PDGF and membrane protein PTK7 in liver cells [53]. This innovative approach leverages the advantages of aptamer synthesis, overcoming issues associated with conventional LYTACs.

The exploration of proteolysis-targeting chimeras (PROTACs) is becoming a promising tools for achieving targeted protein degradation. Nevertheless, the development of drugs utilizing heterobifunctional PROTAC molecules is commonly hindered by challenges such as inadequate membrane permeability, limited in vivo effectiveness, and non-specific distribution. S. He et al. introduces a novel approach to enhance targeted protein degradation using aptamer-proteolysis-targeting chimeras (APCs). The first designed aptamer–PROTAC conjugate (APC) combines a BET-targeting PROTAC with the nucleic acid aptamer AS1411 [54]. This strategy improves tumor-specific targeting, resulting in enhanced in vivo BET degradation and antitumor potency, along with reduced toxicity in an MCF-7 xenograft model. The findings suggest that the aptamer–PROTAC conjugation approach holds promise for developing tumor-specific targeting PROTACs, expanding applications in PROTAC-based drug development.

### 2.4. Aptamer Conjugation with Organic Material

#### 2.4.1. Aptamer–Liposome Conjugation

Liposomes stand out as highly successful drug-delivery systems, with several FDA-approved liposome-based systems for treating diseases in clinical settings [55]. These lipid-based structures have demonstrated the ability to extend the presence of aptamers in the bloodstream. Passive targeting mechanisms, based on the enhanced permeation and retention (EPR) effect for liposomal drug delivery, showed undesirable systemic side effects and suboptimal antitumor effectiveness [55]. The potential enhancement is conceivable through the utilization of delivery vehicles such as aptamers possessing active tumor-targeting capabilities [56,57].

Iman et al. compared the efficacy of nucleolin-targeted PEGylated liposomal doxorubicin (PLD) with PLD in delivering doxorubicin to tumors [58]. Using AS1411 aptamer-coupled liposomes (AS-PLD), the research evaluated cytotoxicity, competition, and cellular uptake in vitro, as well as biodistribution, pharmacokinetics, and therapeutic efficacy in C26 tumor models in mice. The results showed that AS-PLD was more potent in killing cancer cells, exhibited specificity in targeting C26 cells, and demonstrated increased accumulation in tumors after 72 h. The study concludes that AS-PLD is more therapeutically efficient than PLD, making it a suitable active-targeted formulation for cancer treatment.

Han et al. sought to transport miRNA into hepatocellular carcinoma (HCC) cells utilizing liposomes [59]. To boost specificity for HCC cells, the liposomes were altered with the liver cancer-tropic aptamer TLS11a [60]. In mice, liposomes with the aptamer selectively accumulated in the liver area, contrasting with aptamer-free liposomes, which dispersed throughout the body. In this system, the aptamer with liposomes exhibited the utmost delivery efficiency. Khodarahmi et al. studied the clinical use of 5-Fluorouracil (5-FU) for colon cancer, and targeted liposomes were created using an optimized thin film method [61]. The anti-nucleolin aptamer (AS1411) served as a ligand for specific colon targeting. Liposomes were coated with alginate and chitosan to form nanocapsules. Characterization via FT-IR, DLS, zeta potential, and FESEM revealed spherical liposomes (120 nm) and nanocapsules (170 nm). In vitro MTT cytotoxicity studies on the HT-29 colon cancer cell line demonstrated that aptamer-conjugated liposomes induced higher cell death than aptamer-free liposomes and the free drug. Simulated release experiments confirmed the nanocapsules’ efficiency in releasing cargo specifically under colonic conditions. In another study, AS1411 aptamer-conjugated liposomes were employed for the targeted delivery of siRNA against the COL1A1 gene in colorectal cancer (CRC) cells [62]. Cationic liposomes were synthesized, and the confirmation of siRNA loading and aptamer conjugation was achieved through the gel shift assay and spectrophotometry. Cellular studies demonstrated that the liposomal delivery of COL1A1 siRNA into HCT116 and HEK293 cells significantly reduced gene expression, lowered cell viability, increased chemotherapy sensitivity, and induced apoptosis. Aptamer conjugation enhanced these effects in HCT116 cells. The study suggests that the AS1411-targeted liposomal delivery of COL1A1 siRNA is a promising therapeutic strategy for overcoming treatment resistance in CRC.

#### 2.4.2. Aptamer–Micelle Conjugation

Micelles, composed of amphiphilic molecules self-assembled in aqueous solutions, offer significant advantages in drug delivery [63]. The core–shell structure allows for the encapsulation of hydrophobic drugs within the inner core, shielding them from the aqueous environment, while the outer shell provides water solubility. This unique architecture enhances drug stability and bioavailability. Micelles can exploit the enhanced permeability and retention (EPR) effect [64], selectively accumulating in tumor tissues for targeted drug delivery. Additionally, the nanoscale size of micelles facilitates passive targeting and improved cellular uptake. Furthermore, their biocompatibility and ability to incorporate various therapeutic agents make micelles versatile carriers, allowing for the co-delivery of multiple drugs [65] or imaging agents [66], enhancing therapeutic efficacy [63], and personalized medicine strategies [67]. Hence, the assembly of aptamer–micelle conjugates demonstrates significant promise in recognizing cancer cells and has potential applications for in vivo drug delivery [68].

Tian et al. investigated the targeting efficiency and therapeutic efficacy of aptamer-modified polymeric micelles as a drug carrier encapsulating DOX [69]. In vitro cytotoxicity studies revealed enhanced targeting and cytotoxic efficacy against human pancreatic cancer cells (Panc-1 cells) compared to free DOX and DOX-loaded micelles. The aptamer-decorated system exhibits superior tumor penetration into Panc-1 cell spheroids and successful DOX release, suggesting the potential effectiveness of aptamer-modified polymeric micelles for targeted delivery in pancreatic cancer treatment.

The micelle incorporates a targeting aptamer, with the aim of minimizing the therapeutic dosage and reducing off-target effects [68]. The dual redox/pH-sensitive poly (β-amino ester) copolymeric micelles are coupled with the CSRLSLPGSSSKpalmSSS peptide and TA1 aptamer as dual-targeting ligands for synergistic targeting in the 4T1 breast cancer model. A physicochemical characterization confirmed the transformative nature of these stealth nanoparticles (NPs). The micelles, altered into ligand-capped (SRL-2 and TA1) NPs after exposure to the tumor microenvironment, exhibited reduced protein corona formation in Raw 264.7 cells. Notably, the dual-targeted micelles showed a significantly higher accumulation in the 4T1 tumor microenvironment, deep penetration 24 h post-intraperitoneal injection, and remarkable tumor growth inhibition in vivo with a 10% lower therapeutic dose of salinomycin. 

The AS1411 aptamer was studied to develop nucleolar-targeted theranostic pluronic F127-TPGS micelles for brain cancer therapy [70]. Docetaxel (DTX) and upconversion nanoparticles (UCNP) were loaded into micelles, and the TPGS-AS1411 aptamer conjugate was added for brain cancer cell targeting. Micelles were 90–165 nm with a uniform distribution. The DTX and UCNP encapsulation efficiencies were 74–88% and 38–40%, respectively, with sustained DTX release for 72 h. DUTP-AS1411 aptamer micelles were biocompatible and showed a higher effectiveness than Taxotere^®^ in cytotoxicity studies. Brain distribution studies revealed improved efficacy compared to Taxotere^®^, and histopathology studies demonstrated reduced toxicity. The study suggests DUTP-AS1411 aptamer micelles as a promising therapeutic approach for brain cancer with improved efficacy and reduced toxicity.

#### 2.4.3. Aptamer–siRNA Chimeras

RNA interference (RNAi), first observed in *Caenorhabditis elegans*, involves 21- to 23-nucleotide RNA duplexes triggering mRNA degradation [71]. The potential of RNAi for therapeutics was evident when exogenous small interfering RNAs (siRNAs) silenced gene expression in mammalian cells [71]. RNAi’s appealing therapeutic properties include stringent target specificity, low immunogenicity, and simple design. However, a major challenge is delivering siRNAs across cell membranes in vivo. To optimize siRNAs for in vivo use, targeting therapeutic siRNAs to specific cell types is crucial for minimizing side effects and reducing treatment costs. Aptamer–siRNA chimeras offer numerous advantages for in vivo applications [72]. Both aptamers and siRNAs exhibit low immunogenicity, allowing for safe use. They can be produced in large quantities at a cost-effective rate and are adaptable to various chemical modifications, enhancing resistance to degradation and improving in vivo pharmacokinetics. The smaller size of aptamers, in comparison to antibodies, facilitates effective in vivo delivery by enhancing tissue penetration.

A novel RNA therapeutic strategy targeting Osteopontin (OPN) in the tumor microenvironment has been developed by Wei et al. [73]. Recognizing OPN’s role in tumor progression and immune suppression, OPN siRNA was linked to the nucleolin aptamer (Ncl-OPN siRNA) for cancer targeting and connected to the TLR9-binding CpG oligodeoxynucleotide (CpG ODN-OPN siRNA) for myeloid targeting. Treating various cell lines revealed 70–90% OPN inhibition compared to scramble controls, demonstrating therapeutic efficacy in lung and breast cancer cell models. These aptamer–siRNA conjugates show promise as therapeutics with lower toxicity than traditional cytotoxic therapies.

The aptamer–siRNA chimera is used in the development of effective anticancer drugs targeting cancer-associated fibroblasts (CAFs). An aptamer-based conjugate was designed, incorporating a signal transducer and activator of transcription-3 (STAT3) siRNA linked to an aptamer inhibiting platelet-derived growth factor receptor (PDGFRβ). The conjugate effectively delivered STAT3 siRNA to non-small-cell lung cancer (NSCLC) cells, inhibiting CAF-induced cancer cell growth and migration, and reducing spheroid dimension. The conjugate also altered the CAF phenotype, acting as a double agent by inhibiting the entire tumor bulk. This proof-of-principle suggests innovative horizons in NSCLC therapy through aptamer-based siRNA drugs targeting CAF pro-tumor functions.

Utilizing the AS1411 aptamer conjugated with SMG1 RNAi, aptamer-linked siRNA chimeras (AsiCs) were developed to enhance the response to immune-checkpoint blockade (ICB) therapy [74]. AS1411 demonstrated binding to various tumor cell lines and induced cytotoxicity. AS1411-SMG1 AsiCs exhibited a robust antitumor response in local and systemic treatments across different tumor types, improving ICB response. Notably, AS1411-SMG1 AsiCs were well-tolerated with no detected side effects, offering a promising platform to increase the effectiveness of ICB therapy for a broader range of cancer patients.

The development of albumin-binding aptamer–siRNA chimeras was explored to enhance siRNA bioavailability [74]. By fusing RNA-binding aptamers directly to siRNA, these chimeras demonstrated stability in serum, retaining potent gene knockdown capabilities in vitro. In vivo, the best-performing chimera exhibited a 1.6-fold increase in absolute circulation half-life compared to controls. These aptamer–siRNA chimeras effectively improved bioavailability without compromising biological activity, suggesting a promising strategy for drug-delivery applications, particularly for biologic drugs with poor bioavailability.

## 3. Stimuli-Responsive Drug-Delivery Systems for Spatial Anticancer Therapy

As we discussed in the previous section, targetable drug-delivery systems increase drug-delivery efficacy, thus enhancing the anticancer effect. In addition to targetable systems, drug-delivery systems with stimuli-responsive behavior could control the release of drugs spatiotemporally in specific target regions, especially TME and cancer cells. This strategy could increase the delivery efficacy of drugs while reducing the side effects induced by the toxicity of anticancer drugs. Various stimuli, including internal factors such as enzymes, pH level, and reactive oxygen species (ROS), and external factors such as ultrasound, light, and magnetic field, have unique properties individually. Thus, the utilization of transformative behaviors that respond to stimuli could control the drug release at specific regions, resulting in increasing anticancer effects with low side effects. In the following sections, we discuss two parts of stimuli-responsive drug-delivery systems. These sections include discussions of the internal stimuli-responsive drug-delivery system, especially the tumor microenvironment-responsive drug-delivery system, and the external stimuli-responsive drug-delivery system. Furthermore, these sections also discuss smart drug-delivery systems that incorporate active targetable aptamers and stimuli-responsive drug-delivery strategies for synergistic anticancer therapy.

### 3.1. Tumor Microenvironment-Responsive Drug Delivery

Unlike normal tissue, tumor tissue and its microenvironment have special characteristics, such as an acidic pH level, hypoxia conditions, and a high level of certain enzymes and reactive oxygen species [75,76,77,78]. Therefore, the selective transformation of drug-delivery carriers in response to these specific factors in the TME could increase the efficacy of the drug delivery and enhance the anticancer effect.

One promising strategy to increase the drug-delivery efficacy in the TME is the detachment of the polyethylene glycol (PEG) group in response to the TME. PEG is one of the essential substances for the design of drug-delivery carriers to increase colloidal stability with a long nanoparticle circulation time [79,80,81] and to inhibit the absorption of proteins on nanoparticles [80,81,82]. However, PEG on the nanoparticles also could interfere with cell entry, thereby reducing the efficiency of gene and drug delivery against tumor cells [83,84,85]. Therefore, the detachment of PEG from the surface of the nanoparticle in the TME could be useful for increasing the cellular uptake of the carrier, thereby enhancing the therapeutic effects. 

**Figure 3:**
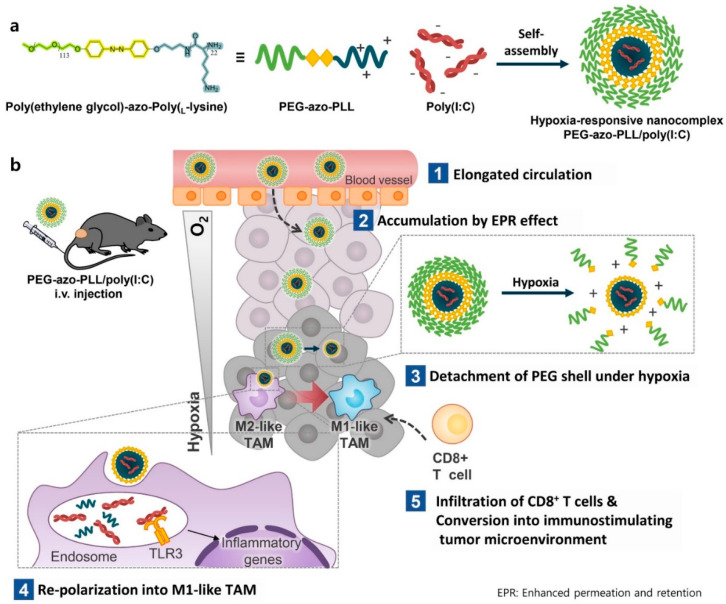
Schematic illustration of the PEG−detachable nanoparticles in the tumor microenvironment for enhanced immunotherapy (Reprinted with permission from ref. [86] and from Elseiver. Copyright 2022, Elsevier). (**a**) Synthetic scheme of hypoxia responsive poly (I:C) delivery nanoparticle. (**b**) Mechanism of immunotherapy in response to TME-sensitive PEG-detaching nanoparticles.

In this regard, Y. Kang et al. recently developed a TME-sensitive PEG-detaching nanoparticle to increase the therapeutic agent delivery for cancer immunotherapy [86] (Figure 3). They developed an azobenzene-conjugated PEG-azo-poly-L-lysine polymer to construct a transformative therapeutic agent carrier. The azobenzene moiety could be degraded by azobenzene reductase under hypoxic conditions [87,88,89], resulting in the detachment of PEG under TME conditions. Polyinosinc:polycytidylic acid (Poly (I:C)), one of the promising therapeutic agents that induce T cell infiltration for immunotherapy [90,91,92], could assemble with PEG-azo-poly L-lysine for constructing nanoparticles by electrostatic interaction. When the nanoparticles were accumulated in the tumor microenvironment, the azobenzene linker that conjugates between PEG and poly (L-lysine) was degraded by azo-reductase and that is upregulated around the tumor microenvironment [93,94,95]. These PEG-detached nanoparticles were highly attracted to the plasma membrane of the surrounding cells, allowing for the efficient delivery of poly (I:C) to tumor-associated macrophages and the stimulation of T cells around the TME, resulting in effective anticancer immunotherapy.

Another strategy to enhance the cellular uptake of drug-delivery nanocarriers is the charge conversion of the nanoparticle in the TME. A cationic-charged surface charge could enhance the cellular uptake of nanoparticles by the electrostatic interaction between the nanoparticle and extracellular membrane, making this strategy mostly used for developing therapeutic agent delivery systems. However, cationic-charged nanoparticles could aggregate with biomolecules such as serum proteins, leading to the decreased efficacy of drug delivery and the induction of uncontrolled inflammatory responses and non-specific cytotoxicity [82,96,97,98,99]. Therefore, reducing the cationic charge on the surface of the nanoparticle during circulation is necessary to inhibit unwanted side effects and increase therapeutic efficacy.

From these points of views, charge conversion nanoparticles, with changeable surface charges from negative to positive in the TME, have several advantages for developing therapeutic agent delivery systems. When these nanoparticles are administrated via the intravenous (IV) route, they are negatively charged, which helps in reducing the adsorption of serum proteins due to electrostatic repulsion and results in less cellular uptake during circulation [100,101,102]. However, when these nanoparticles accumulate in the TME through passive targeting or active targeting, the surface charge of the nanoparticles changes from negative to positive, increasing cellular uptake. This higher cellular uptake could increase the delivery efficacy of anticancer drugs, resulting in enhanced anticancer effects.

**Figure 4:**
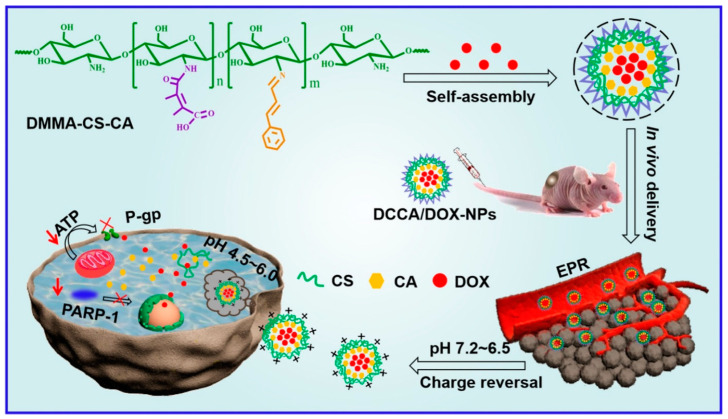
Schematic illustration of the charge conversion system in the TME for anticancer therapy (Reprinted from ref. [103], with permission from Elseiver. Copyright 2022, Elsevier).

Q. Chen et al. developed acidic TME-responsive charge reversal nanoparticles for anticancer drug-delivery systems [103] (Figure 4). Cinnamaldehyde (CA) and 2,3-dimethylmaleic anhydride (DMMA)-modified chitosan (CS) showed amphiphilic properties. Self-assembled nanoparticles were constructed through hydrophobic interaction and enabled the loading of anticancer drugs such as doxorubicin (DOX) by hydrophobic and π–π stacking interactions. In addition, DMMA showed charge reversal properties for selective drug delivery against tumor tissues [104,105]. DMMA conjugated with amino groups exhibited a negative charge during circulation. When the nanoparticle was accumulated in the TME, DMMA could be cleaved in the acidic TME, and the charge was converted to a positive charge. This positive charge could enhance drug delivery against tumor cells. 

**Figure 5:**
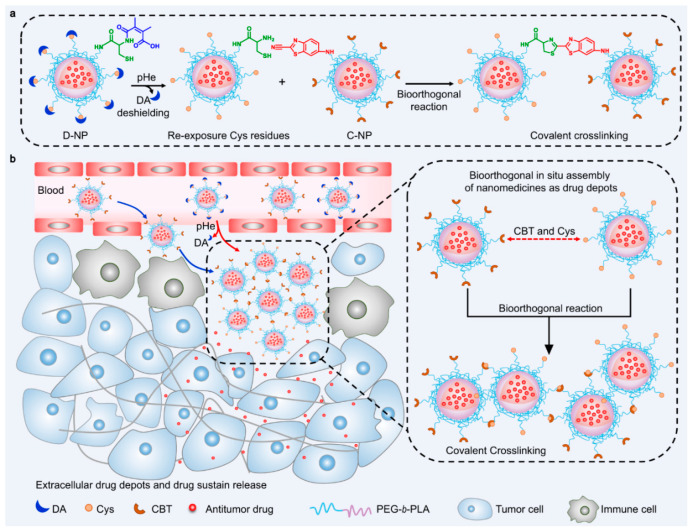
Schematic illustration of crosslinked nanoparticle system in TME for enhanced antitumor activity (Reprinted with permission from ref. [106] under the terms of the Creative Commons CC BY license. Copyright 2022, Springer Nature). (**a**) Schematic illustration of the bioorthogonal reaction under TME. (**b**) Mechanism of prolonged drug release when D-NPs and C-NPs were co-administrated in the TME.

Another strategy for enhancing the efficacy of drug-delivery systems is to assemble the accumulated drug-delivery vehicles in response to internal stimuli within the TME [106,107,108,109]. The assembly of the drug-delivery vehicle could prolong the retention time of therapeutic drugs in the TME, resulting in the enhancement of therapeutic efficacy. From this perspective, Z. Cao et al. developed crosslinked drug-delivery nanoparticles through a cycloaddition reaction in the TME [106]. To execute this strategy, they designed two distinct types of drug-delivery nanoparticles, the DMMA-Cysteine-modified polymeric nanoparticle and cyanobenzothiazole (CBT)-modified polymeric nanoparticle. When these nanoparticles were co-accumulated in the TME, the DMMA was detached from the DMMA-Cys modified nanoparticle due to the acidic condition of the TME. The exposed cysteine residue could react with the CBT-modified polymeric nanoparticles by a bioorthogonal cycloaddition reaction. This reaction causes the two types of nanoparticles to crosslink with each other, forming microparticle formations. (Figure 5) This strategy can increase the retention time of the drug-delivery vehicle with a prolonged drug release, ensuring prolonged drug release and improving the delivery efficacy with anti-metastatic efficacy. In addition, this strategy also has the potential to deliver various types of anticancer drugs, allowing for a combination of chemotherapy and immunotherapy.

**Figure 6:**
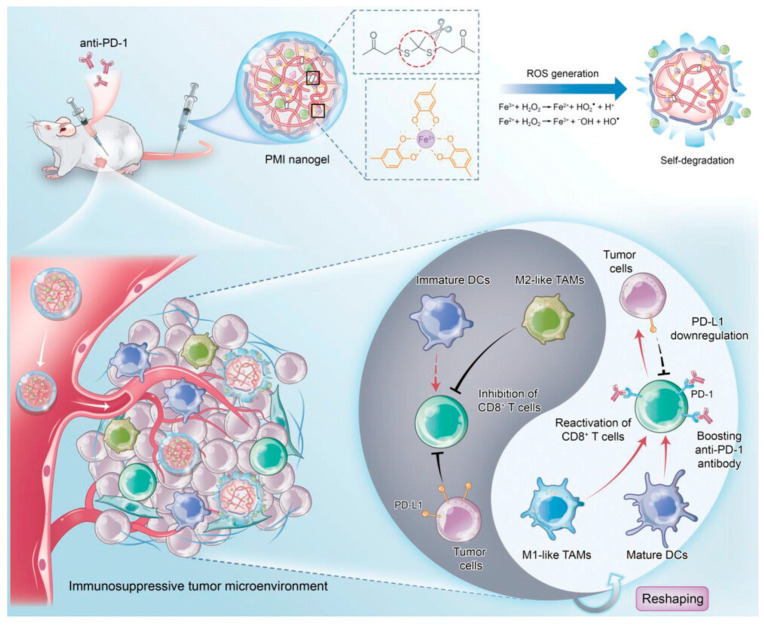
ROS−responsive drug delivery system for anticancer immunotherapy (Reprinted with permission from ref. [112] under the terms of the Creative Commons CC BY license. Copyright 2023, John Wiley and Sons).

In addition to the assembled system, therapeutic systems based on the strategies that can maximize drug delivery through the selective disruption of drug carriers within the TME have been developed. Together with the acidic condition at the tumor site, the elevation of the levels of reactive oxygen species (ROS) is one of the specific properties of the TME [78,110,111]. Therefore, ROS-responsive self-degradable nanoparticles could deliver anticancer drugs selectively at the tumor site. For example, H. Tian et al. developed phenolic polymer-based ROS-responsive nanogels for cancer immunotherapy [112] (Figure 6). The crosslinked nanogel, constructed by a thioketal linker and metal–phenolic coordination, could be rapidly disrupted via elevated ROS levels surrounding the TME, together with an iron-mediated catalytic reaction [113,114,115,116]. This selective and rapid disruption of the nanogel could sufficiently release immune modulators such as metformin and imiquimod [117,118,119,120,121], resulting in the activation of T cell-mediated anticancer effects.

### 3.2. External Stimuli-Responsive Drug Delivery

#### 3.2.1. Ultrasound-Mediated Drug-Delivery Systems

Ultrasound (US) techniques, with acoustic wave frequencies greater than 20 kHz, have been widely used for biomedical applications such as imaging and therapy [122,123,124,125]. Due to their mechanical and thermal effects [122,123,124], several ultrasound-mediated drug-delivery systems have been recently explored for effective anticancer therapy. 

**Figure 7:**
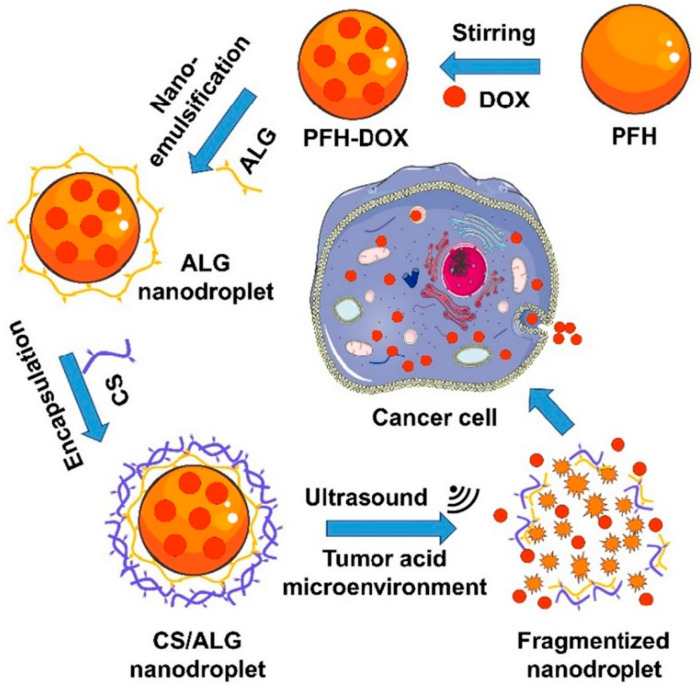
Preparation of the core–shell nanodroplet for spatiotemporal anticancer drug delivery at tumor tissue (Reprinted from ref [126], with permission from Elsevier. Copyright 2021, Elsevier).

Y. Gao described the nanodroplet-loaded nanoparticle for US-mediated drug delivery against cancer cells [126] (Figure 7). To construct this system, they developed a core–shell nanoparticle structure. The core consists of perfluorocarbone (PFH), which is vaporized and transformed into microbubbles through the application of ultrasound [127,128]. This bubbling can collapse the nanoparticle, inducing an accelerated anticancer drug release in the PFH core when exposed to US. To stabilize the nanodroplet under physiological conditions, biocompatible biopolymers such as alginate (ALG) and chitosan (CS) were used to formulate the anticancer drug-delivering nanodroplet. When this nanoparticle was administrated and accumulated at the tumor site, DOX was released in a spatiotemporal manner, dependent on the US irradiation around the tumor tissue. Additionally, the electrostatic interaction between CS and ALG was weakened under acidic conditions [129], resulting in accelerated drug release in the acidic tumor microenvironment. Therefore, this system is useful for spatiotemporal and site-specific drug delivery for effective anticancer therapy.

**Figure 8:**
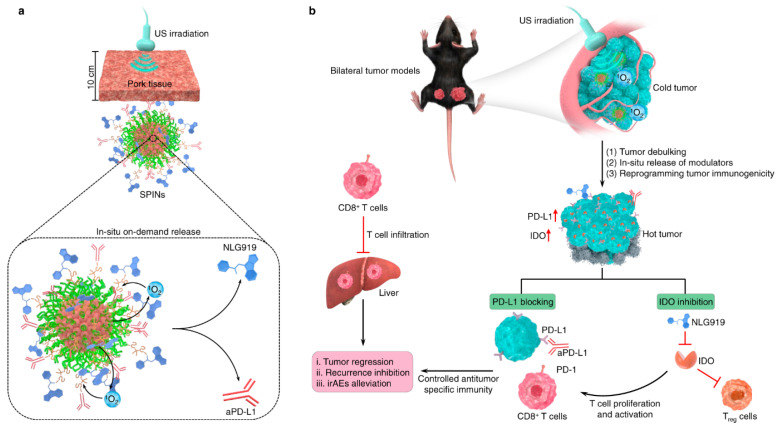
US-mediated ROS generation for synergistic anticancer therapy combined with sonodynamic ROS-mediated therapy with accelerated release of immunotherapeutic agents (Reprinted with permission from ref. [130] under the terms of the Creative Commons CC BY license. Copyright 2022, Springer Nature). (**a**) Schematic illustration of the release of NLG919 and anti−PD−L1 antibody from sonosensitizer−loaded US−responsive nanoparticle. (**b**) Mechanism of US-mediated anticancer therapy under US irradiation with US−responsive nanoparticles.

In addition to the nanodroplet-based anticancer drug-delivery system, it was also possible to construct the US-mediated drug-delivery system with a sonosensitizer and reactive oxygen species (ROS)-mediated cleavage linker [130] (Figure 8). The sonosensitizer could produce ROS during US irradiation [131,132], resulting in the cleavage of the ROS-responsive chemical linkers, such as the thioketal linker, and the thioester and arylboronic ester linker [115,116,133,134]. In addition, this sonodynamically activated ROS could induce cancer cell death; thus, ROS generation could be useful for not only accelerating drug release but also therapeutic efficacy [131,132,135,136,137]. 

From this point of view, J. Li et al. developed a sonosensitizer-loaded nanoparticle for US sonodynamic anticancer therapy with an accelerated release of immunotherapeutic agents in the tumor microenvironment [130]. Through the sonosensitizer screening process to find a high efficacy of ROS generation with a high stability under repeated US irradiation, it was found that the SP7-conjugated polymer, consisting of benzothiadiazole and dialkoxybenzodithiophene [138,139], could generate high levels of ROS reliably. This proper sonosensitizer was then formulated with a poly-(ethylene glycol)-block-poly-(propylene glycol)-block- poly-(ethylene glycol) amphiphilic triblock copolymer for the construction of a water-dispersible nanoparticle. This nanoparticle was then conjugated with immunotherapeutic agents, such as the NLG919 and anti-PD-L1 antibody, along with an ROS-cleavable thioketal linker. NLG919, a potent inhibitor of indoleamine 2,30dioxygenase-1 (IDO)1, could inhibit IDO-mediated immune suppression and activate CD8+ T cells for the regression of tumor tissue [140,141]. Anti PD-L1 antibodies are FDA-approved immune checkpoint inhibitors that activate the antitumor immunity of T cells [142,143]. Therefore, this nanoparticle was highly useful for effective anticancer therapy, accompanying the ROS-mediated anticancer agents and site-specific release of immunotherapeutic agents at the proper time upon US irradiation. 

In addition to the cases discussed above, heat generated by focused ultrasound has been used for spatiotemporal drug release. High intensity-focused ultrasound (HIFU) techniques that localize high intensity (100–10,000 W/cm^2^) frequencies in the focal area, generate thermal effects exceeding 40 °C; thus, they have been recently applied to treat various kinds of solid tumors using thermal ablation [144,145,146]. In addition to the thermal ablation of cancer cells, this thermal effect could rapidly increase the release of anticancer drugs in the TME, resulting in a synergistic effect for effective anticancer therapy.

**Figure 9:**
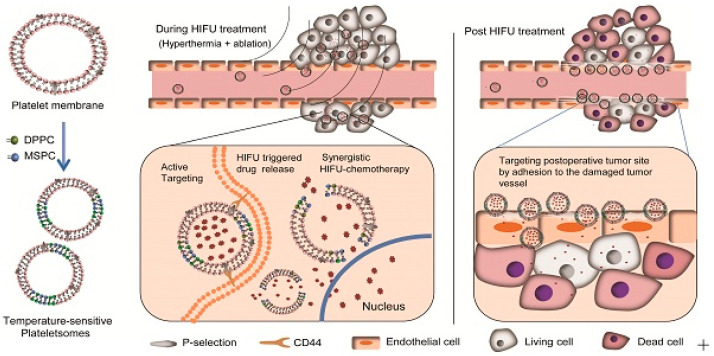
Thermal-sensitive drug-delivery system for synergistic anticancer therapy (Reprinted from ref. [147]. Copyright 2019, Ivyspring International Publisher).

From this point of view, D. Wu et al. developed a thermal-sensitive drug-delivery carrier for an HIFU-mediated combination therapy [147] (Figure 9). They prepared platelet membrane-based nanovesicles, which serve to prolong blood circulation time with cancer-targeting effects [148,149,150,151,152]. In addition to the platelet membrane (PM), two types of lipids, such as 1-stearoyl-2-hydroxy-sn-glycero-3-phosphocholine (MSPC) and 1,2-dipalmitoyl-sn-glycero-3-phosphocholine (DPPC), were employed in this vesicle to endow it with thermal-sensitive drug-release properties [153,154]. When the drug-loaded nanovesicles were administrated with HIFU irradiation in the TME, these nanovesicles were highly accumulated in the TME and experienced a burst release of anticancer drugs in response to HIFU irradiation, resulting in a synergistic effect between thermal ablation and chemotherapy.

**Figure 10:**
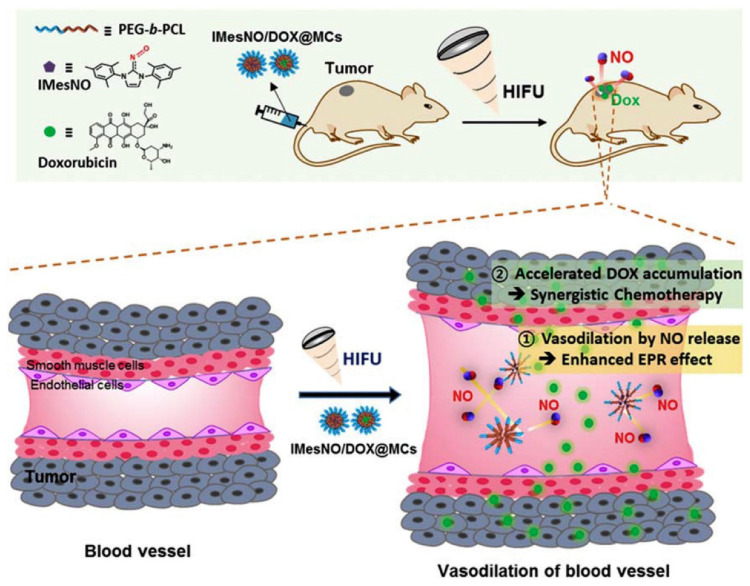
Schematic illustration of HIFU-mediated therapeutic agent delivery system for enhanced anticancer therapy. HIFU generated NO at tumor tissue, which accelerated DOX accumulation to increase anticancer effect (Reprinted from ref. [155], with permission from Elseiver. Copyright 2019, Elsevier).

The localized temperature increase not only induces the structural change of the drug-delivery carrier but also decomposes certain thermally labile chemical bonds. Therefore, HIFU techniques have also been applied to cleave bonds for localized therapeutic agent delivery. For example, Y. Kang et al. developed a HIFU-mediated nitric oxide release for enhanced anticancer therapy [155] (Figure 10). Nitric oxides (NOs) are gaseous radical molecules that regulate biological activities such as angiogenesis, vasodilation, and anticancer related modulation, depending on the concentration [156,157,158]. Among the various functions of NO, the additional vasodilation and angiogenesis in the TME can enhance the anticancer effects due to the increase of drug accumulation within the TME [159,160,161]. Therefore, the HIFU-mediated NO releasing system with anticancer drugs could induce the enhanced drug accumulation in the TME spatiotemporally in response to HIFU irradiation. 

To prepare this system, N-hetrocyclic carbine (NHC)-based NO donors (NHC-NOs), which are stable at room temperature and generate NO at a high temperature through the thermolysis of the chemical bonds between NHC and NO, was used [155,162]. Therefore, this NO donor generates NO spatiotemporally against HIFU, resulting in the selective NO generation in the TME with reduced side effects. When NHC-NO-loaded micelles and anticancer drug doxorubicin (DOX)-loaded micelles were co-administrated with HIFU irradiation against tumor tissues, HIFU-mediated NO could induce vasodilation, resulting in a greater accumulation of DOX-loaded micelles and thereby increasing the anticancer effects.

#### 3.2.2. Photo-Responsive Nanoparticle Drug-Delivery Systems for Anticancer Therapy

Due to its noninvasiveness, simple manipulation, high spatial resolution and spatiotemporal controllability, light has been widely used for biomedical applications [163,164,165]. Within the diverse spectrum of light, near-infrared (NIR) light (wavelength range 700–1400 mm) has found extensive use in both diagnostic and therapeutic applications [164,165,166,167]. This is attributed to its lower photo-toxicity and ability to penetrate deep into tissues with minimal absorption [166,167,168]. In addition, the integration of NIR light with photo-active materials, such as photothermal agents, photodynamic sensitizers, and upconversion nanoparticles [169,170,171,172,173,174,175,176], has been recently employed to develop photo-responsive drug-delivery systems for anticancer therapy.

One promising strategy for developing photo-responsive drug-delivery systems involves manipulating NIR light with photothermal agents. Photothermal agents could absorb the energy of NIR light and convert it to heat energy, generating localized heat at the site of light irradiation [174,175,176]. This localized heat can alter the structure or phase of the thermo-sensitive materials, resulting in the rapid release of cargo from thermo-sensitive material-based delivery carrier upon light irradiation [177,178,179,180,181]. 

In addition to achieving a spatiotemporal drug release, photothermal effects can induce heat shock responses against tumor cells [166,176], resulting in the thermal ablation of tumor cells [182,183,184,185]. Therefore, the combination of thermo-sensitive materials with photothermal agents have been applied for multifunctional drug-delivery systems for synergistic anticancer therapy.

**Figure 11:**
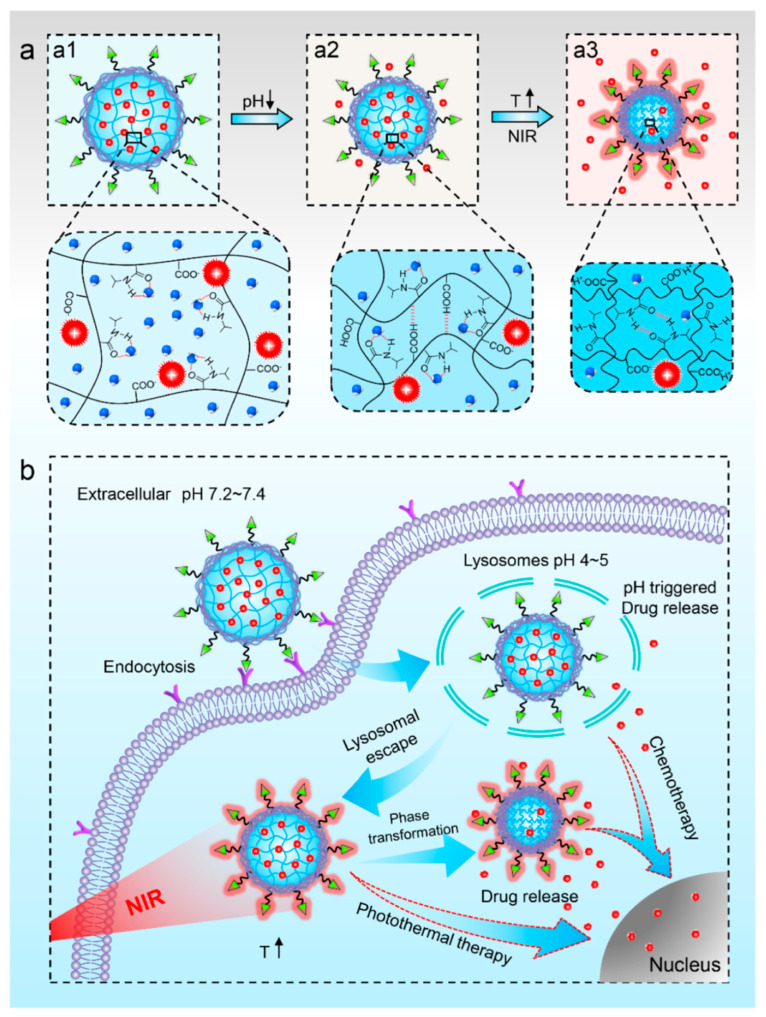
(**a**) Schematic illustration of the nanoparticle with pH−responsive (**a1**,**a2**) and NIR−responsive (**a2**,**a3**) drug release characteristics. (**b**) Schematic illustration of the synergetic chemo−photothermal therapy for tumor cell with the nanoparticles (Reprinted with permission from ref. [186]. Copyright 2021, American Chemical Society).

X.-Q. Pu et al. developed a multifunctional photo-responsive nanoparticle for chemo–photothermal therapy in cancer treatment [186] (Figure 11). This nanoparticle consists of a poly (*N*-isopropylacrylamide-co-acrylic acid) (PNA) nanogel core and a folic acid-conjugated polydopamine (PDA) shell. The PNA nanogel core obtained a poly (N- isopropylacrylamide) (PNIPAM) group that shows thermo-sensitive phase transition properties [177,187,188]. The anticancer drug doxorubicin (DOX) was loaded in the PNA nanogel via electrostatic interaction [186,189,190]. This drug exhibited an accelerated release under low pH conditions, occurring during the endocytosis process in cancer cells [186,189,191,192]. The outer PDA shell exhibited photothermal activity [193,194,195], accelerating drug release due to the phase transition of the PNA nanogel core and inducing photothermal tumor ablation. Additionally, folic acids conjugated on the PDA shell demonstrated targeting activity against tumor cells [196,197]. Therefore, this developed nanoparticle exhibited the selective delivery of anticancer drugs spatiotemporally due to its active targeting ability and photo-induced release properties, in addition to photo-induced cancer tissue ablation for enabling effectively synergistic anticancer therapy. 

The photothermal activity not only changes the phase of the polymeric nanoparticle, but also regulates nucleic acid dynamics for the spatiotemporal release of drugs. Nucleic acids, such as deoxyribonucleic acid (DNA) and ribonucleic acid (RNA), hybridize with complementary strands and this hybridization could load anticancer drugs via intercalation [198,199,200]. However, with an increase in temperature, the hybridized structure could be degenerated, resulting in the release of loaded drugs [201,202]. Therefore, the combination of nucleic acids with photothermal agents has been applied to construct photo-responsive drug-delivery systems. 

**Figure 12:**
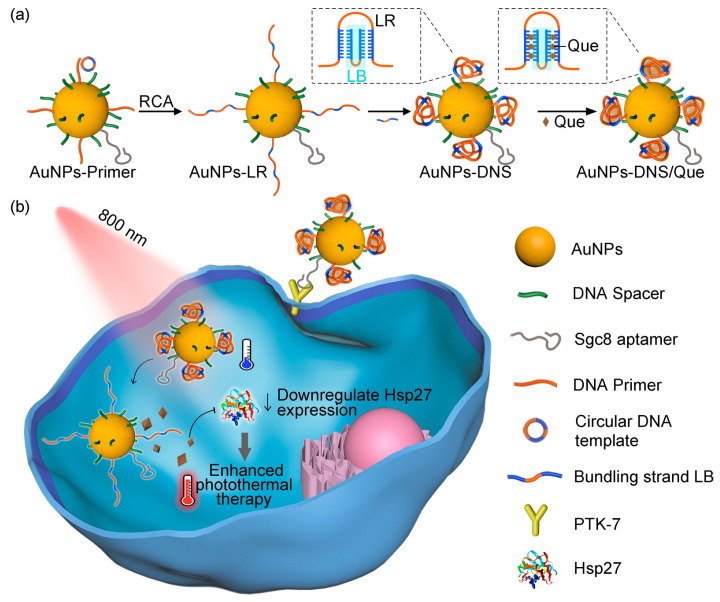
Schematic illustration of photothermal-induced DNA dynamics for anticancer therapy. (**a**) Scheme of DNA-incorporated gold nanoparticle with Que loading. (**b**) Mechanistic illustration of anticancer effect of nanoparticle upon light irradiation (Reprinted with permission from ref. [203] under the terms of the Creative Commons CC BY license. Copyright 2023, Springer Nature).

Recently, Y. Yang et al. developed a DNA-based drug-delivery carrier modified with gold nanoparticles for an effective photo-mediated anticancer therapy [203] (Figure 12). Long strand DNAs were decorated on gold nanoparticles in situ using rolling circle amplification, followed by hybridization with complementary DNA strands on the gold nanoparticles. These hybridization structures could load quercetin (Que) as a heat shock protein inhibitor, increasing the efficacy of thermal therapy [204,205]. When the NIR light was irradiated in the TME after these nanoparticles were accumulated, Que was spatiotemporally released in the TME upon light irradiation due to the photothermal effect of gold nanoparticles. This Que inhibits the heat shock protein, resulting in enhanced photothermal effects and effective anticancer therapy. 

In addition to the use of photothermal activity, photodynamic activity could be applied for the spatiotemporal release of drugs against tumor tissues. Photodynamic activity involves the generation of ROS through light irradiation in the presence of the photosensitizing agents (PSs) and oxygen [171,206,207,208,209]. This ROS could cleave ROS-labile linkers, resulting in the decomposition of the drug-delivery carrier to rapidly release the drugs. In addition, photo-triggered ROS generation initiates anticancer therapy, which is known as photodynamic therapy (PDT). Therefore, ROS-labile linkers incorporating drug-delivery carriers with PSs and anticancer drugs have been applied for synergistic anticancer therapy. 

**Figure 13:**
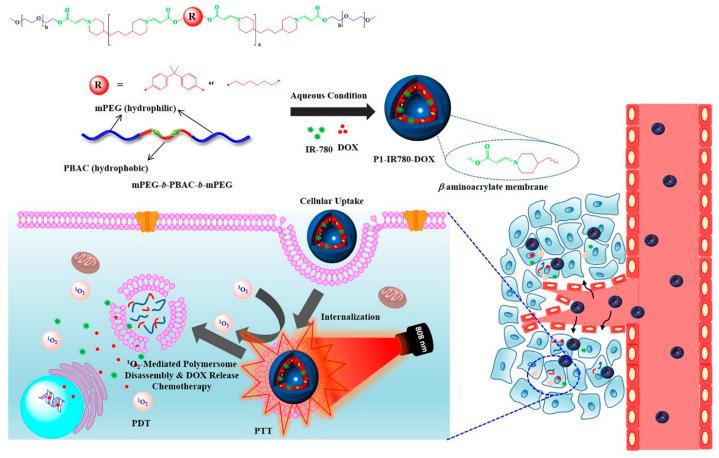
Schematic illustration of ROS-responsive polymersomes for ROS-mediated drug delivery with PDT (Reprinted from ref. [210], with permission from Elsevier. Copyright 2020, Elsevier).

Recently, G. Saravanakumar et al. developed ROS-responsive polymersomes for NIR-controlled combined chemo-phototherapy [210] (Figure 13). To prepare the ROS-responsive polymersomes, they synthesized amphiphilic polymers, including hydrophilic polyethylene glycol segments and hydrophobic poly (β-aminoacrylate) segments with aromatic or aliphatic backbone structures, via amino-alkynoate click polymerization [210,211,212]. These amphiphilic polymers were self-assembled in aqueous conditions to construct polymersomes. When anticancer drugs such as DOX and PSs such as IR-780 were co-incorporated in polymersomes and accumulated in tumor tissue, DOXs were rapidly released via ROS generation by PSs in response to light irradiation. In addition, the generation of ROS induces PDT, resulting in synergistic anticancer therapy combining chemotherapy and PDT.

Beyond harnessing photothermal and photodynamic effects, ongoing efforts are focused on advancing a system that optimizes drug delivery through the utilization of upconversion nanoparticles (UCNPs). UCNPs convert NIR light to high energy UV-VIS light upon NIR light irradiation for imaging and therapeutic applications [169,213,214]. This system has several advantages over the direct irradiation of UV-VIS light. Although UV-VIS light has high energy that is suitable for cleaving photo-labile linkers such as nitrobenzyl-based linkers, the penetration depth of UV light is too low for applications in deep tissue [169,215,216,217]. Additionally, UV light exhibited photo-toxicity and carcinogenic properties, limiting its clinical applications. In comparison, NIR light has a greater depth of penetration, but the energy of the light is too low to cleave certain bonds by itself. Therefore, a system that combines UCNPs and NIR light harnesses the advantages of both UV-VIS light and NIR light, resulting in the construction of a photo-responsive drug-delivery system without lethal photo-toxicity.

**Figure 14:**
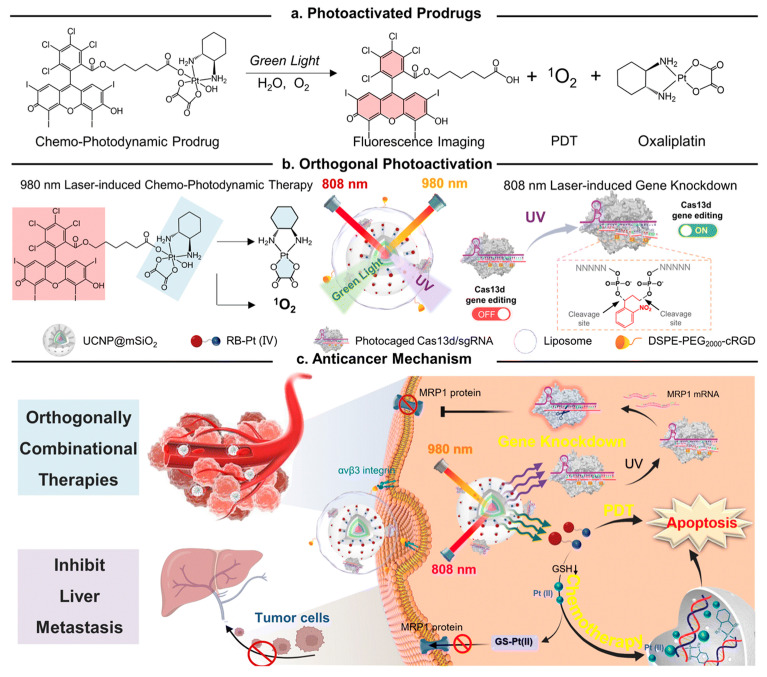
Schematic illustration depicting the incorporation of the GA-prodrug and UA-RNA in UCNPs for NIR-activatable synergistic anticancer therapy (Reprinted from ref. [218]. Copyright 2023, Royal Society of Chemistry).

From this perspective, Z. Liu et al. have developed a UCNP-based drug-delivery system for synergistic anticancer therapy [218] (Figure 14). To develop an advanced therapeutic system, they synthesized a green light-activatable anticancer prodrug and UV light activatable gene–gene editing sequences. The green light-activatable prodrug (GA-prodrug) material, conjugated with PSs such as Rose Bengal and anticancer drugs such as oxaliplatin, generate ROS with oxaliplatin in response to green light irradiation. The UV light-activatable gene editing RNAs (UA-RNA), hybridized with single-guided RNA (sgRNA) and UV-labile linker-modified complementary sequences, were dehybridized upon UV irradiation, activating the CRISPR/Cas13d system to cleave multidrug resistance-associated protein 1 (MRP1) mRNA, resulting in the downregulation of multi-drug resistance (MDR) in tumor cells and increased sensitization to chemo drugs. These two photo-responsive drugs and genes were incorporated with core-multi-shell UCNPs that convert 808 nm and 980 nm of light to UV and green light, respectively. The converted UV light and green light were activated with GA-prodrugs and UA-RNA, respectively, delivering anticancer drugs and the MDR-downregulating CRISPR/Cas13d system to enhance anticancer effects. In addition, PSs in GA-prodrugs generate ROS during light irradiation, resulting in the synergistic effects of anticancer treatment due to PDT.

#### 3.2.3. Magnetic Field-Responsive Nanomaterials for Anticancer Therapy

Magnetic fields have been manipulated in biomedical fields; for example, magnetic resolution image (MRI) and magnetic hyperthermia techniques are used for anticancer therapy [219,220,221,222]. In addition, this magnetic field guidance has been employed to direct magnetic nanoparticles, including superparamagnetic iron oxide nanoparticles (SPIONs), toward specific regions. This process improves the accumulation of drugs at target regions such as the TME [223,224,225]. Therefore, magnetic field-responsive nanomaterials have garnered attention for developing advanced anticancer therapeutic systems.

**Figure 15:**
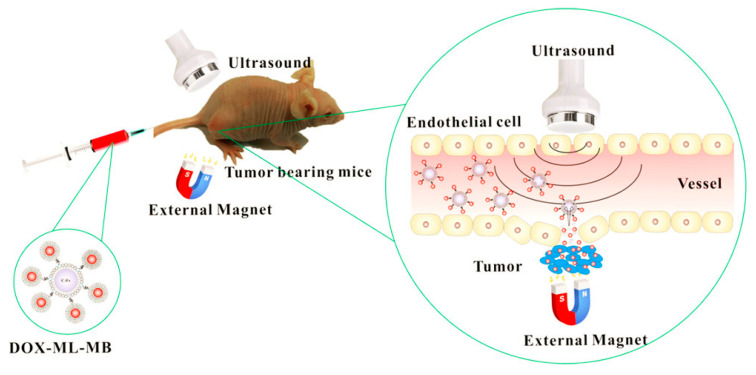
Schematic illustration of microbubbles incorporating MNPs for a dual stimuli-responsive drug-delivery system, responsive to both a magnetic field and ultrasound (Reprinted with permission from ref. [226]. Copyright 2020, American Chemical Society).

One promising strategy is to produce magnetic field-guided drug-delivery systems, where magnetic nanoparticles are directed by magnetic fields. By controlling the magnetic field with drug-loaded magnetic nanoparticles (MNPs), the accumulation of these nanoparticles can be concentrated toward target tissues, resulting in the construction of a targeted and efficient drug-delivery system. Recently, P. Dwivedi et al. developed magnetic-guided nanoparticles and incorporated ultrasound(US)-responsive microbubbles for enhanced anticancer therapy [226] (Figure 15). Fe_3_O_4_ MNPs and DOX were encapsulated in liposomes, and these nanoparticles were conjugated with microbubbles to construct dual stimuli-responsive microparticles. Upon administration, these microparticles deeply penetrated the targeted tumor tissue in response to the controlled magnetic field. Furthermore, US also deeply penetrated tumor tissues and decomposed the microparticles via US-mediated microbubble vaporization, resulting in an effective drug release deep within the tumor tissue. Therefore, these two stimuli-responsive drug-delivery systems have the potential for cancer therapy applications against deep tumor tissues such as pancreatic cancer.

Another strategy for the magnetic-responsive drug-delivery system involves utilizing magnetic hyperthermia (MH) activity [220,221,227]. MH activity occurs when MNPs are accumulated in the target region in response to the alternating magnetic field (AMF). The MNPs absorbed the energy of the AMF and converted the energy into a large amount of heat during magnetization. This heat can elevate the local temperature above 43 °C, leading to the thermal ablation of target tissues. 

Additionally, it can alter the shape or decompose nanoparticles, resulting in the rapid release of cargo upon AMF irradiation [227,228]. Therefore, MNPs with an AMF can be applied to construct a system that utilizes hyperthermia-mediated anticancer therapy, accelerating anticancer drug release for synergistic anticancer therapy.

**Figure 16:**
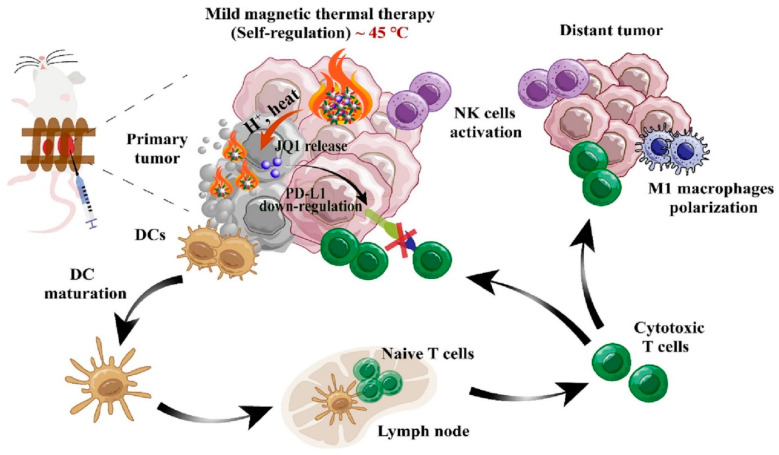
An overall strategy involves the utilization of MH-responsive nano-assembled MNPs for synergistic anticancer therapy, combining magnetic-guided therapy with immunotherapy (Reprinted from ref. [228], with permission from Elsevier. Copyright 2023, Elsevier).

Recently, M. Gao et al. developed MH-mediated drug-delivery system combined with immunotherapy for effective cancer therapy [228] (Figure 16). To promote better MH activity, ~10 nm of monodispersed nanoparticles were crosslinked via a temperature-responsive chemical linker to ~100 nm of MNPs. After constructing the nano-assembly, the immune-stimulating bromodomain and ester-terminal domain inhibitor JQ1 were loaded into the nano-assembled MNPs for immunotherapy [228,229,230,231,232]. Upon the accumulation of these nanoparticles against tumor tissue with an AMF, MH activity exhibited both MH-based tumor ablation and the rapid release of JQ1 via the thermally-induced degradation of the chemical linker [228,233,234]. In addition, mild heating from MH activity could create a fever-like heat to induce an immune-favorable microenvironment to stimulate immunotherapy [228,235,236]. The MH-mediated release of JQ1 downregulates PD-L1 and sensitizes immunotherapy. Therefore, the administrated nano-assembled MNPs exhibited high antitumor effects, demonstrating the synergistic effect between MH-mediated antitumor activity and immunotherapy. 

### 3.3. Smart Drug Delivery Targeted with the Aptamer

The field of drug-delivery systems has changed with the emergence of more intelligent approaches to targeted cancer treatment [237]. Smart drug-delivery systems incorporating stimuli responsiveness, based on chemical structure modifications triggered by internal or external stimuli, are considered the future of drug delivery. Consequently, smart delivery holds promise as a potential alternative for cancer treatment, moving beyond conventional chemotherapy. This section involves a discussion of smart drug-delivery systems, particularly those incorporating aptamers.

One example of an aptamer-based smart drug-delivery system involves manipulating the transformable aptamer in response to internal stimuli. Internal stimuli, such as adenosine-5′-triphosphate (ATP), could interact with specific aptamers containing ATP-binding motifs, causing a change in their secondary or tertiary structure, and thereby accelerating drug release [238]. Importantly, the concentration of ATP in intracellular conditions is much higher than in the extracellular environment [239]. Therefore, the manipulation of aptamers that possess both tumor-receptor-binding ability and ATP-binding motifs could exhibit both tumor targeting and spatial drug release against intra-tumor cells. Mo et al. employed adenosine-5′-triphosphate (ATP) as a trigger for controlled anticancer drug release [239]. Polymeric nanocarriers, incorporating an ATP-binding aptamer in the DNA motif, selectively releases doxorubicin in ATP-rich environments via a conformational switch. The ATP-responsive nanovehicles exhibit a 3.6-fold increase in cytotoxicity in MDA-MB-231 cells compared to non-ATP-responsive nanovehicles. With a hyaluronic acid-crosslinked outer shell for specific tumor targeting, these nanocarriers demonstrate the enhanced chemotherapeutic inhibition of tumor growth in xenograft MDA-MB-231 tumor-bearing mice. This ATP-triggered drug release system offers a sophisticated approach, selectively releasing drugs based on ATP levels. In another study, Mo et al. introduced a novel ATP-responsive anticancer drug-delivery strategy utilizing DNA-graphene hybrid nanoaggregates [240]. ATP, acting as the trigger, responds to the intracellular ATP concentration, leading to the enhanced release of preloaded drugs. The hybrid nanoaggregates, composed of GO, single-stranded DNA (DNA1 and DNA2), and an ATP aptamer, effectively control the release of DOX. In the presence of ATP, the formation of the ATP/ATP aptamer complex prompts the dissociation of the aggregates, facilitating targeted and on-demand drug delivery within specific cells.

The utilization of additional internal stimuli-responsive drug-delivery carriers, accompanied by an ATP-responsive aptamer, has also been developed for enhanced anticancer therapy [241]. M. Zhao et al. developed an aptamer-based dual internal stimuli-responsive drug-delivery system for combinatory anticancer therapy. The DNA scaffolds in this system contain a tumor-targeting AS1411 aptamer, ATP aptamer, and its complementary DNA sequence for both loading the anticancer drug DOX and achieving a tumor-targeting effect. These DNA scaffolds selectively release DOX in intra-tumor conditions in response to ATP. Additionally, these scaffolds were employed against manganese dioxide (MnO_2_) nanosheets via a coordinated interaction for constructing a dual stimuli-responsive drug-delivery system. When DNA scaffold-embedded MnO_2_ nanosheets were accumulated in cancer cells, the overexpression of glutathione (GSH) in the tumor cells reduced MnO_2_ and broke its structure, resulting in the release of DNA scaffolds from the nanosheets and thereby accelerating drug release [241]. In fact, it was confirmed that drug release occurs more quickly when ATP and GSH exist together compared to when ATP or GSH exist alone. 

Alongside the internal stimuli-responsive delivery system, an external stimuli-responsive smart drug-delivery system that incorporates aptamers has also been developed recently. Near-infrared (NIR)-activatable tumor-targeting delivery systems allowed controllable cancer-cell binding for potentially specific and efficient cancer therapy. Yang et al. presents a light-activatable cancer-targeting strategy using a complementary DNA sequence to mask aptamers on photothermal agents like gold nanorods or single-walled carbon nanotubes (SWNTs) [242]. The NIR laser exposure, along with localized photothermal heating, causes the dehybridization of the DNA, unveiling the aptamer for specific cancer-cell targeting. Using doxorubicin-loaded SWNTs as a model, targeted drug delivery to cancer cells activated by NIR light has been achieved. Zhang et al. developed a novel nanocarrier that is responsive to NIR light, using Cu1.8S nanoparticles coated with mesoporous silica [243]. The nanoparticles, with a high photothermal conversion efficiency, were selected for cancer thermotherapy and on-demand drug release. The mesoporous silica structure was used for drug storage/delivery and was modified with aptamer-modified DNA-helix gatekeepers, drug vectors, and targeting ligands. The NIR-responsive nanocomposites exhibited a synergistic therapeutic effect by releasing hydrophilic drug doxorubicin and hydrophobic drug curcumin upon NIR irradiation, demonstrating significant growth inhibition and apoptosis in cancer cells. This multifunctional nanoplatform holds promise for effective cancer therapy. 

Cai et al. developed a novel drug-delivery system utilizing molybdenum disulfide(MoS_2_) nanosheets, known for their high photothermal conversion efficiency, enabling the targeted chemo–photothermal treatment of cancer cells [244]. Loaded with the chemotherapy DOX and coated with polydopamine (PDA), the MoS_2_ nanosheets are modified with a thiolated aptamer AS1411 and polyethylene glycol (PEG) to construct DOX@Apt-PEG-PDA-MoS_2_ nanosheets. The nanoplatform, endowed with the ability to target breast cancer cells, exhibits good biocompatibility, and achieves synergetic chemo–photothermal therapy effects with enhanced antitumor efficacy, making it a promising drug-delivery platform for targeted and comprehensive cancer treatment.

Mesoporous polydopamine (MPDA) is known for loading chemotherapy agents, enhancing anticancer therapy, and minimizing side effects [245]. Furthermore, it demonstrates strong near-infrared (NIR) absorption, effectively converting NIR light into heat to eliminate cancer cells. This capability enables the synergistic treatment of cancers through combined chemotherapy and photothermal therapy. Dai et al. developed multifunctional nanoparticles, AS1411@MPDA-DTX (AMD), for targeted and synergistic chemotherapy/photothermal therapy in prostate cancer [246]. The nanoparticles demonstrated the efficient targeting of prostate cancer cells, facilitated the internalization of DTX, and enhanced the antitumor effects during chemo–photothermal therapy under NIR laser irradiation, as evidenced by both in vitro and in vivo results.

In addition to using photothermal activity, photo-responsive cleavable strategies have also been employed in the construction of smart drug-delivery systems. For example, Yang et al. developed a novel photo-responsive hyperbranched polymer grafted with DNA aptamers for precise and controlled drug delivery [247]. The constructed nanoparticles exhibit biocompatibility, target specificity, and light-controllable release behavior. Under UV irradiation, the rapid release of Nile Red-loaded nanoparticles occurs through disassembly. In vitro cell studies confirm the system’s specific binding and internalization facilitated by the DNA aptamer corona. The doxorubicin-loaded nano-assembly demonstrates selective photo-triggered cytotoxicity against cancer cells, showcasing its potential as a “smart” drug-delivery system with promising therapeutic effects.

Another strategy for constructing smart drug-delivery systems using external stimuli responsiveness is to employ magnetic-guided tumor therapy. The induction of an AMF with magnetic nanoparticles could exhibit MH activity, leading to thermal tumor therapy and accelerated drug release. Therefore, the manipulation of tumor-targetable aptamers with magnetic nanoparticles contributes to the development of smart drug-delivery system. Z. Chen et al. developed aptamer-incorporated zinc-doped iron oxide nano-octahedrons for smart anticancer therapy [248]. The tumor-targetable AS1411 guided the increased accumulation of nanocarriers against tumor cells, enhancing drug-delivery efficacy and MH-based anticancer effects. Furthermore, when the AMF was applied around tumor tissue, these nanocarriers could rapidly release the anticancer drug DOX. Together with accelerated drug release, MH activities applied with silencing heat shock proteins, such as HSP70/HSP90, could induce synergistic anticancer therapy through the combination of MH-mediated anticancer effects and chemotherapy.

## 4. Future Perspectives and Limitations

The future of utilizing aptamers in drug delivery for cancer therapy presents an exciting landscape filled with promise and innovation. As we look ahead, one can anticipate a surge in research aimed at refining aptamer-selection techniques, including advancements in SELEX methodologies, to yield aptamers with high specificity and affinity for an array of cancer biomarkers. The integration of these optimized aptamers into sophisticated drug-delivery systems holds the potential to revolutionize cancer treatment by enabling unprecedented precision and personalized approaches. The concept of aptamer-based theranostics [249], where these molecules serve a dual role as both targeting agents and diagnostic tools, emerges as a transformative avenue for the real-time monitoring of treatment response. However, amidst these optimistic prospects, several critical limitations loom on the horizon. 

The stability of aptamers in biological environments is a hurdle, requiring comprehensive research into modifications that enhance resistance to nucleases and other degrading agents [249]. In addition, the lack of diversity in the aptamer library is another drawback [250], which refers to a potential limitation in the repertoire of aptamer candidates available for selection against various targets. Moreover, concerns regarding off-target interactions [251] and unintended side effects underline the critical importance of rigorous preclinical studies and comprehensive safety assessments. 

The future of aptamer-based drug-delivery systems for cancer therapy hinges on overcoming these challenges [252]. Ongoing efforts to enhance aptamer technology will likely involve interdisciplinary collaborations, incorporating insights from materials science, nanotechnology, and bioengineering [253]. Advances in aptamer modifications, bioconjugation techniques, and delivery systems will be pivotal in addressing the aforementioned limitations. 

The integration of artificial intelligence and computational modeling may further expedite the design and optimization of aptamers for specific therapeutic applications, facilitating a more targeted and streamlined drug-development process [254]. As we navigate the path toward the clinical realization of aptamer-based cancer therapies, a holistic understanding of the interplay between aptamers and biological systems, coupled with a commitment to safety and efficacy, will guide the translation of these groundbreaking innovations into transformative clinical interventions. The future holds great promise for aptamer-based drug-delivery systems in cancer therapy, but the journey forward requires a concerted and multidisciplinary effort to surmount the obstacles that currently stand in its way. 

Moreover, the successful commercialization of drug-delivery technologies mentioned above remains a challenge. Initially, it is essential to establish a system that ensures the consistent management of the characteristics of developed products during mass production and batch-manufacturing processes. Although targeted and stimulus-sensitive drug-delivery systems have been successfully developed in laboratory settings, for widespread commercial application, the technology must advance to produce these systems at a kilogram scale. Furthermore, effective management strategies and validated product-evaluation methods for various targetable moieties, as well as qualification and quantification abilities, are essential. Strategies ensuring uniform sizes in mass-produced nanoparticles, along with measures for maintaining stimulus sensitivity uniformity and regulation, are also imperative. 

Additionally, a toxicity evaluation of decomposition products generated after drug delivery is necessary. Following the implementation of the drug-delivery system, a pre-clinical toxicity test, such as acute/repeatable/genetic toxicity in a Good Laboratory Practice (GLP) environment, is required to assess whether the remaining by-products affect other normal organs in the body or cause toxicity. Moreover, the relationship between preclinical testing and clinical testing must be clearly identified to ensure that there are no toxicity issues, and effectiveness is improved when actual clinical trials are conducted. Finally, it is necessary to develop more advanced and high-resolution equipment for an improved drug-delivery system using external stimulation.

## 5. Conclusions

As discussed in previous sections, smart targeting and spatial trigger-responsive drug-delivery systems have been widely developed for effective anticancer therapy. Particularly, nanoparticles have demonstrated tumor-specific accumulation due to the enhanced permeability and retention (EPR) effect, making them attractive for advanced anticancer therapy. In addition to the passive tumor-targeting ability of nanoparticles, manipulating active targeting moieties such as antibodies and aptamers guides drug-delivery carriers more effectively against cancer cells, increasing efficacy and anticancer effects. Among these targeting moieties, we specifically discussed aptamer-based drug-delivery systems due to their advantages, such as low toxicity, negligible immunogenicity, and versatility applied to various types of nanoparticles. 

Moreover, we have also discussed spatial stimuli-responsive drug-delivery systems. These systems control the release of anticancer drugs spatiotemporally against tumor microenvironments, resulting in the increased specificity of chemotherapy and reduced side effects. Therefore, these strategies have valuable potential for patient-favorable anticancer treatments. The stimuli, including internal factors like enzymes, pH, or ROS, and external factors such as ultrasound, light, or magnetism, have been manipulated to construct stimuli-responsive drug-delivery systems. While these targeting and spatial drug-delivery systems currently face challenges such as long-term stability, mass production, and the evaluation of the toxicity of the by-products after implementation, overcoming these hurdles will pave the way for the feasible development of system-based medicines in the future, advancing anticancer treatments with the potential to enhance anticancer treatment, making it more patient-friendly by improving drug efficacy and selectivity. Moreover, with the recent commercialization of nanoparticle-based medicines like mRNA vaccines, the development of medicines using these systems will become feasible upon the advancement of mass-production technology and product-management methods.

## References

[1] Ellington A.D., Szostak J.W. (1990). In vitro selection of RNA molecules that bind specific ligands. Nature.

[2] Tuerk C., Gold L. (1990). Systematic evolution of ligands by exponential enrichment: RNA ligands to bacteriophage T4 DNA polymerase. Science.

[3] Alshaer W., Hillaireau H., Fattal E. (2018). Aptamer-guided nanomedicines for anticancer drug delivery. Adv. Drug Deliv. Rev..

[4] Choi J.W., Seo M., Kim K., Kim A.R., Lee H., Kim H.S., Park C.G., Cho S.W., Kang J.H., Joo J. (2023). Aptamer Nanoconstructs Crossing Human Blood-Brain Barrier Discovered via Microphysiological System-Based SELEX Technology. ACS Nano.

[5] Nguyen T.T.Q., Kim E.R., Gu M.B. (2022). A new cognate aptamer pair-based sandwich-type electrochemical biosensor for sensitive detection of Staphylococcus aureus. Biosens. Bioelectron..

[6] Wan L.Y., Yuan W.F., Ai W.B., Ai Y.W., Wang J.J., Chu L.Y., Zhang Y.Q., Wu J.F. (2019). An exploration of aptamer internalization mechanisms and their applications in drug delivery. Expert Opin. Drug Deliv..

[7] Röthlisberger P., Hollenstein M. (2018). Aptamer chemistry. Adv. Drug Deliv. Rev..

[8] Fu Z., Xiang J. (2020). Aptamers, the nucleic acid antibodies, in cancer therapy. Int. J. Mol. Sci..

[9] Zeng Z., Qi J., Wan Q., Zu Y. (2021). Aptamers with Self-Loading Drug Payload and pH-Controlled Drug Release for Targeted Chemotherapy. Pharmaceutics.

[10] Zhang Y., Zhang H., Chan D.W.H., Ma Y., Lu A., Yu S., Zhang B., Zhang G. (2022). Strategies for developing long-lasting therapeutic nucleic acid aptamer targeting circulating protein: The present and the future. Front. Cell Dev. Biol..

[11] Vazquez-Gonzalez M., Willner I. (2021). Aptamer-Functionalized Micro- And Nanocarriers for Controlled Release. ACS Appl. Mater. Interfaces.

[12] Li S.L., Jiang P., Jiang F.L., Liu Y. (2021). Recent Advances in Nanomaterial-Based Nanoplatforms for Chemodynamic Cancer Therapy. Adv. Funct. Mater..

[13] Mahmoudpour M., Ding S., Lyu Z., Ebrahimi G., Du D., Ezzati Nazhad Dolatabadi J., Torbati M., Lin Y. (2021). Aptamer functionalized nanomaterials for biomedical applications: Recent advances and new horizons. Nano Today.

[14] Shiao Y.S., Chiu H.H., Wu P.H., Huang Y.F. (2014). Aptamer-functionalized gold nanoparticles as photoresponsive nanoplatform for Co-drug delivery. ACS Appl. Mater. Interfaces.

[15] Yang X., Yang M., Pang B., Vara M., Xia Y. (2015). Gold Nanomaterials at Work in Biomedicine. Chem. Rev..

[16] Lv Q.-Y., Cui H.-F., Song X. (2023). Aptamer-based technology for gastric cancer theranostics. Anal. Methods.

[17] Elghanian R., Storhoff J.J., Mucic R.C., Letsinger R.L., Mirkin C.A. (1997). Selective Colorimetric Detection of Polynucleotides Based on the Distance-Dependent Optical Properties of Gold Nanoparticles. Science.

[18] Hu R., Wen W., Wang Q., Xiong H., Zhang X., Gu H., Wang S. (2014). Novel electrochemical aptamer biosensor based on an enzyme–gold nanoparticle dual label for the ultrasensitive detection of epithelial tumour marker MUC1. Biosens. Bioelectron..

[19] Yang L., Tseng Y.-T., Suo G., Chen L., Yu J., Chiu W.-J., Huang C.-C., Lin C.-H. (2015). Photothermal Therapeutic Response of Cancer Cells to Aptamer–Gold Nanoparticle-Hybridized Graphene Oxide under NIR Illumination. ACS Appl. Mater. Interfaces.

[20] Dou B., Xu L., Jiang B., Yuan R., Xiang Y. (2019). Aptamer-Functionalized and Gold Nanoparticle Array-Decorated Magnetic Graphene Nanosheets Enable Multiplexed and Sensitive Electrochemical Detection of Rare Circulating Tumor Cells in Whole Blood. Anal. Chem..

[21] Khorshid M., Varshosaz J., Rostami M., Haghiralsadat F., Akbari V., Khorshid P. (2023). Anti HER-2 aptamer functionalized gold nanoparticles of dasatinib for targeted chemo-radiotherapy in breast cancer cells. Biomater. Adv..

[22] Navyatha B., Nara S. (2023). Aptamer- and antibody-conjugated gold nanobipyramids—A study on cytotoxicity towards breast cancer cell lines. J. Nanoparticle Res..

[23] Manzano M., Vallet-Regí M. (2020). Mesoporous Silica Nanoparticles for Drug Delivery. Adv. Funct. Mater..

[24] Fu Z., Xiang J. (2020). Aptamer-Functionalized Nanoparticles in Targeted Delivery and Cancer Therapy. Int. J. Mol. Sci..

[25] Vandghanooni S., Barar J., Eskandani M., Omidi Y. (2020). Aptamer-conjugated mesoporous silica nanoparticles for simultaneous imaging and therapy of cancer. TrAC Trends Anal. Chem..

[26] Heydari S.R., Ghahremani M.H., Atyabi F., Bafkary R., Jaafari M.R., Dinarvand R. (2023). Aptamer-modified chitosan-capped mesoporous silica nanoparticles for co-delivery of cytarabine and daunorubicin in leukemia. Int. J. Pharm..

[27] Kianpour M., Huang C.-W., Vejvisithsakul P.P., Wang J.-Y., Li C.-F., Shiao M.-S., Pan C.-T., Shiue Y.-L. (2023). Aptamer/doxorubicin-conjugated nanoparticles target membranous CEMIP2 in colorectal cancer. Int. J. Biol. Macromol..

[28] Xie X., Li F., Zhang H., Lu Y., Lian S., Lin H., Gao Y., Jia L. (2016). EpCAM aptamer-functionalized mesoporous silica nanoparticles for efficient colon cancer cell-targeted drug delivery. Eur. J. Pharm. Sci..

[29] Cui X., Xu S., Wang X., Chen C. (2018). The nano-bio interaction and biomedical applications of carbon nanomaterials. Carbon.

[30] Kościk I., Jankowski D., Jagusiak A. (2021). Carbon Nanomaterials for Theranostic Use. C.

[31] Yaghoubi F., Naghib S.M., Motlagh N.S.H., Haghiralsadat F., Jaliani H.Z., Tofighi D., Moradi A. (2021). Multiresponsive carboxylated graphene oxide-grafted aptamer as a multifunctional nanocarrier for targeted delivery of chemotherapeutics and bioactive compounds in cancer therapy. Nanotechnol. Rev..

[32] Viraka Nellore B.P., Pramanik A., Chavva S.R., Sinha S.S., Robinson C., Fan Z., Kanchanapally R., Grennell J., Weaver I., Hamme A.T. (2014). Aptamer-conjugated theranostic hybrid graphene oxide with highly selective biosensing and combined therapy capability. Faraday Discuss..

[33] Zhao C., Song X., Jin W., Wu F., Zhang Q., Zhang M., Zhou N., Shen J. (2019). Image-guided cancer therapy using aptamer-functionalized cross-linked magnetic-responsive Fe_3_O_4_@carbon nanoparticles. Anal. Chim. Acta.

[34] Zavareh H.S., Pourmadadi M., Moradi A., Yazdian F., Omidi M. (2020). Chitosan/carbon quantum dot/aptamer complex as a potential anticancer drug delivery system towards the release of 5-fluorouracil. Int. J. Biol. Macromol..

[35] Biju V. (2014). Chemical modifications and bioconjugate reactions of nanomaterials for sensing, imaging, drug delivery and therapy. Chem. Soc. Rev..

[36] Liu Q., Xu L., Zhang X., Li N., Zheng J., Guan M., Fang X., Wang C., Shu C. (2013). Enhanced Photodynamic Efficiency of an Aptamer-Guided Fullerene Photosensitizer toward Tumor Cells. Chem. Asian J..

[37] Jha R., Singh A., Sharma P.K., Fuloria N.K. (2020). Smart carbon nanotubes for drug delivery system: A comprehensive study. J. Drug Deliv. Sci. Technol..

[38] Chen W., Yang S., Wei X., Yang Z., Liu D., Pu X., He S., Zhang Y. (2020). Construction of Aptamer-siRNA Chimera/PEI/5-FU/Carbon Nanotube/Collagen Membranes for the Treatment of Peritoneal Dissemination of Drug-Resistant Gastric Cancer. Adv. Healthc. Mater..

[39] Liu J., Cui L., Losic D. (2013). Graphene and graphene oxide as new nanocarriers for drug delivery applications. Acta Biomater..

[40] Shahidi M., Haghiralsadat B.F., Abazari O., Hemati M., Dayati P., Jaliani H.Z., Motlagh N.S.H., Naghib S.M., Moradi A. (2023). HB5 aptamer-tagged graphene oxide for co-delivery of doxorubicin and silibinin, and highly effective combination therapy in breast cancer. Cancer Nanotechnol..

[41] Liu Z., Robinson J.T., Sun X., Dai H. (2008). PEGylated Nanographene Oxide for Delivery of Water-Insoluble Cancer Drugs. J. Am. Chem. Soc..

[42] Lu J., Zhang A., Zhang F., Linhardt R.J., Zhu Z., Yang Y., Zhang T., Lin Z., Zhang S., Zhao H. (2023). Ganoderenic acid D-loaded functionalized graphene oxide-based carrier for active targeting therapy of cervical carcinoma. Biomed. Pharmacother..

[43] Baneshi M., Dadfarnia S., Haji Shabani A.M., Sabbagh S.K., Bardania H. (2022). AS1411 aptamer-functionalized graphene oxide-based nano-carrier for active-target and pH-sensitive delivery of curcumin. J. Iran. Chem. Soc..

[44] He J., Duan Q., Ran C., Fu T., Liu Y., Tan W. (2023). Recent progress of aptamer–drug conjugates in cancer therapy. Acta Pharm. Sin. B.

[45] Xuan W., Peng Y., Deng Z., Peng T., Kuai H., Li Y., He J., Jin C., Liu Y., Wang R. (2018). A basic insight into aptamer-drug conjugates (ApDCs). Biomaterials.

[46] Powell Gray B., Kelly L., Ahrens D.P., Barry A.P., Kratschmer C., Levy M., Sullenger B.A. (2018). Tunable cytotoxic aptamer–drug conjugates for the treatment of prostate cancer. Proc. Natl. Acad. Sci. USA.

[47] Sicco E., Cerecetto H., Calzada V., Moreno M. (2023). Targeted-Lymphoma Drug Delivery System Based on the Sgc8-c Aptamer. Cancers.

[48] Zhang N., Wang J., Bing T., Liu X., Shangguan D. (2022). Transferrin receptor-mediated internalization and intracellular fate of conjugates of a DNA aptamer. Mol. Ther. Nucleic Acids.

[49] Henri J.L., Nakhjavani M., McCoombe S., Shigdar S. (2023). Cytotoxic effects of aptamer-doxorubicin conjugates in an ovarian cancer cell line. Biochimie.

[50] Ma W., Yang Y., Liu Z., Zhao R., Wan Q., Chen X., Tang B., Zhou Y., Lin Y. (2023). Self-Assembled Multivalent Aptamer Drug Conjugates: Enhanced Targeting and Cytotoxicity for HER2-Positive Gastric Cancer. ACS Appl. Mater. Interfaces.

[51] Jo J., Bae S., Jeon J., Youn H., Lee G., Ban C. (2023). Bifunctional G-Quadruplex Aptamer Targeting Nucleolin and Topoisomerase 1: Antiproliferative Activity and Synergistic Effect of Conjugated Drugs. Bioconjug. Chem..

[52] Liu B., Wang J., Peng Y., Zeng H., Zhang Q., Deng M., Xiang W., Liu J., Hu X., Liu X. (2023). CD71/CD44 dual-aptamer-gemcitabine conjugate for tumor co-targeting treatment of bladder cancer. Chem. Eng. J..

[53] Wu Y., Lin B., Lu Y., Li L., Deng K., Zhang S., Zhang H., Yang C., Zhu Z. (2023). Aptamer-LYTACs for Targeted Degradation of Extracellular and Membrane Proteins. Angew. Chemie Int. Ed..

[54] He S., Gao F., Ma J., Ma H., Dong G., Sheng C. (2021). Aptamer-PROTAC Conjugates (APCs) for Tumor-Specific Targeting in Breast Cancer. Angew. Chemie Int. Ed..

[55] Woo J., Chiu G.N.C., Karlsson G., Wasan E., Ickenstein L., Edwards K., Bally M.B. (2008). Use of a passive equilibration methodology to encapsulate cisplatin into preformed thermosensitive liposomes. Int. J. Pharm..

[56] Jain Singhai N., Maheshwari R., Khatri K. (2023). New insights in aptamer-targeted nanoliposomes for the treatment of breast cancer. J. Drug Deliv. Sci. Technol..

[57] Moosavian S.A., Kesharwani P., Singh V., Sahebkar A. (2023). 6-Aptamer-functionalized liposomes for targeted cancer therapy. Woodhead Publishing Series in Biomaterials.

[58] Iman M., Moosavian S.A., Zamani P., Jaafari M.R. (2023). Preparation of AS1411 aptamer-modified PEGylated liposomal doxorubicin and evaluation of its anti-cancer effects in vitro and in vivo. J. Drug Deliv. Sci. Technol..

[59] Han S., Xue L., Wei Y., Yong T., Jia W., Qi Y., Luo Y., Liang J., Wen J., Bie N. (2023). Bone Lesion-Derived Extracellular Vesicles Fuel Prometastatic Cascades in Hepatocellular Carcinoma by Transferring ALKBH5-Targeting miR-3190-5p. Adv. Sci..

[60] Hu Z., He J., Gong W., Zhou N., Zhou S., Lai Z., Zheng R., Wang Y., Yang X., Yang W. (2018). TLS11a Aptamer/CD3 Antibody Anti-Tumor System for Liver Cancer. J. Biomed. Nanotechnol..

[61] Khodarahmi M., Abbasi H., Kouchak M., Mahdavinia M., Handali S., Rahbar N. (2022). Nanoencapsulation of aptamer-functionalized 5-Fluorouracil liposomes using alginate/chitosan complex as a novel targeting strategy for colon-specific drug delivery. J. Drug Deliv. Sci. Technol..

[62] Manoochehri H., Jalali A., Tanzadehpanah H., Taherkhani A., Najafi R. (2022). Aptamer-conjugated nanoliposomes containing COL1A1 siRNA sensitize CRC cells to conventional chemotherapeutic drugs. Colloids Surf. B Biointerfaces.

[63] Alven S., Aderibigbe B.A. (2020). The Therapeutic Efficacy of Dendrimer and Micelle Formulations for Breast Cancer Treatment. Pharmaceutics.

[64] Biancacci I., De Lorenzi F., Theek B., Bai X., May J.-N., Consolino L., Baues M., Moeckel D., Gremse F., von Stillfried S. (2022). Monitoring EPR Effect Dynamics during Nanotaxane Treatment with Theranostic Polymeric Micelles. Adv. Sci..

[65] Cho H., Lai T.C., Tomoda K., Kwon G.S. (2015). Polymeric micelles for multi-drug delivery in cancer. AAPS PharmSciTech.

[66] Vorobiev V., Adriouach S., Crowe L.A., Lenglet S., Thomas A., Chauvin A.-S., Allémann E. (2021). Vascular-targeted micelles as a specific MRI contrast agent for molecular imaging of fibrin clots and cancer cells. Eur. J. Pharm. Biopharm..

[67] Prencipe F., Diaferia C., Rossi F., Ronga L., Tesauro D. (2021). Forward Precision Medicine: Micelles for Active Targeting Driven by Peptides. Molecules.

[68] Salahpour-Anarjan F., Zare F., Hosseini F., Ahranjani S.D., Alipour M., Gozali E. (2023). 7-Aptamer-functionalized micelles for targeted cancer therapy. Woodhead Publishing Series in Biomaterials.

[69] Tian L., Pei R., Zhong L., Ji Y., Zhou D., Zhou S. (2021). Enhanced targeting of 3D pancreatic cancer spheroids by aptamer-conjugated polymeric micelles with deep tumor penetration. Eur. J. Pharmacol..

[70] Chauhan M., Singh R.P., Yadav B., Shekhar S., Kumar A., Mehata A.K., Nayak A.K., Dutt R., Garg V., Kailashiya V. (2023). Development and characterization of micelles for nucleolin-targeted co-delivery of docetaxel and upconversion nanoparticles for theranostic applications in brain cancer therapy. J. Drug Deliv. Sci. Technol..

[71] Cao Z., Tong R., Mishra A., Xu W., Wong G.C.L., Cheng J., Lu Y. (2009). Reversible Cell-Specific Drug Delivery with Aptamer-Functionalized. Angew. Chem. Int. Ed..

[72] McNamara J.O., Andrechek E.R., Wang Y., Viles K.D., Rempel R.E., Gilboa E., Sullenger B.A., Giangrande P.H. (2006). Cell type–specific delivery of siRNAs with aptamer-siRNA chimeras. Nat. Biotechnol..

[73] Wei J., Song R., Sabbagh A., Marisetty A., Shukla N., Fang D., Najem H., Ott M., Long J., Zhai L. (2022). Cell-directed aptamer therapeutic targeting for cancers including those within the central nervous system. Oncoimmunology.

[74] Zhang L., Mu C., Zhang T., Wang Y., Wang Y., Fan L., Liu C., Chen H., Shen J., Wei K. (2020). Systemic Delivery of Aptamer-Conjugated XBP1 siRNA Nanoparticles for Efficient Suppression of HER2+ Breast Cancer. ACS Appl. Mater. Interfaces.

[75] Uthaman S., Huh K.M., Park I.-K. (2018). Tumor microenvironment-responsive nanoparticles for cancer theragnostic applications. Biomater. Res..

[76] Jin M.-Z., Jin W.-L. (2020). The updated landscape of tumor microenvironment and drug repurposing. Signal Transduct. Target. Ther..

[77] Zhou F., Fu T., Huang Q., Kuai H., Mo L., Liu H., Wang Q., Peng Y., Han D., Zhao Z. (2019). Hypoxia-Activated PEGylated Conditional Aptamer/Antibody for Cancer Imaging with Improved Specificity. J. Am. Chem. Soc..

[78] Weinberg F., Ramnath N., Nagrath D. (2019). Reactive Oxygen Species in the Tumor Microenvironment: An Overview. Cancers.

[79] Knop K., Hoogenboom R., Fischer D., Schubert U.S. (2010). Poly(ethylene glycol) in Drug Delivery: Pros and Cons as Well as Potential Alternatives. Angew. Chemie Int. Ed..

[80] Suk J.S., Xu Q., Kim N., Hanes J., Ensign L.M. (2016). PEGylation as a strategy for improving nanoparticle-based drug and gene delivery. Adv. Drug Deliv. Rev..

[81] D’souza A.A., Shegokar R. (2016). Polyethylene glycol (PEG): A versatile polymer for pharmaceutical applications. Expert Opin. Drug Deliv..

[82] Rausch K., Reuter A., Fischer K., Schmidt M. (2010). Evaluation of Nanoparticle Aggregation in Human Blood Serum. Biomacromolecules.

[83] Song L.Y., Ahkong Q.F., Rong Q., Wang Z., Ansell S., Hope M.J., Mui B. (2002). Characterization of the inhibitory effect of PEG-lipid conjugates on the intracellular delivery of plasmid and antisense DNA mediated by cationic lipid liposomes. Biochim. Biophys. Acta BBA.

[84] Mishra S., Webster P., Davis M.E. (2004). PEGylation significantly affects cellular uptake and intracellular trafficking of non-viral gene delivery particles. Eur. J. Cell Biol..

[85] Mok H., Bae K.H., Ahn C.-H., Park T.G. (2009). PEGylated and MMP-2 Specifically DePEGylated Quantum Dots: Comparative Evaluation of Cellular Uptake. Langmuir.

[86] Kang Y., Lim J., Saravanakumar G., Kim J., Park M., Im S., Kim W.J. (2022). Immunostimulation of tumor microenvironment by targeting tumor-associated macrophages with hypoxia-responsive nanocomplex for enhanced anti-tumor therapy. J. Control. Release.

[87] Kulkarni P., Haldar M.K., You S., Choi Y., Mallik S. (2016). Hypoxia-Responsive Polymersomes for Drug Delivery to Hypoxic Pancreatic Cancer Cells. Biomacromolecules.

[88] Piao W., Hanaoka K., Fujisawa T., Takeuchi S., Komatsu T., Ueno T., Terai T., Tahara T., Nagano T., Urano Y. (2017). Development of an Azo-Based Photosensitizer Activated under Mild Hypoxia for Photodynamic Therapy. J. Am. Chem. Soc..

[89] Im S., Lee J., Park D., Park A., Kim Y.-M., Kim W.J. (2019). Hypoxia-Triggered Transforming Immunomodulator for Cancer Immunotherapy via Photodynamically Enhanced Antigen Presentation of Dendritic Cell. ACS Nano.

[90] Aznar M.A., Planelles L., Perez-Olivares M., Molina C., Garasa S., Etxeberría I., Perez G., Rodriguez I., Bolaños E., Lopez-Casas P. (2019). Immunotherapeutic effects of intratumoral nanoplexed poly I:C. J. Immunother. Cancer.

[91] Sultan H., Wu J., Fesenkova V.I., Fan A.E., Addis D., Salazar A.M., Celis E. (2020). Poly-IC enhances the effectiveness of cancer immunotherapy by promoting T cell tumor infiltration. J. Immunother. Cancer.

[92] Du X., Hou Y., Huang J., Pang Y., Ruan C., Wu W., Xu C., Zhang H., Yin L., He W. (2021). Cytosolic delivery of the immunological adjuvant Poly I:C and cytotoxic drug crystals via a carrier-free strategy significantly amplifies immune response. Acta Pharm. Sin. B.

[93] Sharma A., Arambula J.F., Koo S., Kumar R., Singh H., Sessler J.L., Kim J.S. (2019). Hypoxia-targeted drug delivery. Chem. Soc. Rev..

[94] Park H., Saravanakumar G., Kim J., Lim J., Kim W.J. (2021). Tumor Microenvironment Sensitive Nanocarriers for Bioimaging and Therapeutics. Adv. Healthc. Mater..

[95] Lee S., Kim Y., Lee E.S. (2022). Hypoxia-Responsive Azobenzene-Linked Hyaluronate Dot Particles for Photodynamic Tumor Therapy. Pharmaceutics.

[96] Merhi M., Dombu C.Y., Brient A., Chang J., Platel A., Le Curieux F., Marzin D., Nesslany F., Betbeder D. (2012). Study of serum interaction with a cationic nanoparticle: Implications for in vitro endocytosis, cytotoxicity and genotoxicity. Int. J. Pharm..

[97] Wei X., Shao B., He Z., Ye T., Luo M., Sang Y., Liang X., Wang W., Luo S., Yang S. (2015). Cationic nanocarriers induce cell necrosis through impairment of Na^+^/K^+^-ATPase and cause subsequent inflammatory response. Cell Res..

[98] Wen M., Li Y., Zhong W., Li Q., Cao L., Tan L.-L., Shang L. (2022). Interactions of cationic gold nanoclusters with serum proteins and effects on their cellular responses. J. Colloid Interface Sci..

[99] Goodman C.M., McCusker C.D., Yilmaz T., Rotello V.M. (2004). Toxicity of Gold Nanoparticles Functionalized with Cationic and Anionic Side Chains. Bioconjugate Chem..

[100] Nandhakumar S., Dhanaraju M.D., Sundar V.D., Heera B. (2017). Influence of surface charge on the in vitro protein adsorption and cell cytotoxicity of paclitaxel loaded poly(ε-caprolactone) nanoparticles. Bull. Fac. Pharm. Cairo Univ..

[101] Zhao J., Wu S., Qin J., Shi D., Wang Y. (2018). Electrical-Charge-Mediated Cancer Cell Targeting via Protein Corona-Decorated Superparamagnetic Nanoparticles in a Simulated Physiological Environment. ACS Appl. Mater. Interfaces.

[102] Augustine R., Hasan A., Primavera R., Wilson R.J., Thakor A.S., Kevadiya B.D. (2020). Cellular uptake and retention of nanoparticles: Insights on particle properties and interaction with cellular components. Mater. Today Commun..

[103] Chen Q., Jia C., Xu Y., Jiang Z., Hu T., Li C., Cheng X. (2022). Dual-pH responsive chitosan nanoparticles for improving in vivo drugs delivery and chemoresistance in breast cancer. Carbohydr. Polym..

[104] Du J.-Z., Li H.-J., Wang J. (2018). Tumor-Acidity-Cleavable Maleic Acid Amide (TACMAA): A Powerful Tool for Designing Smart Nanoparticles To Overcome Delivery Barriers in Cancer Nanomedicine. Acc. Chem. Res..

[105] Zhang P., Chen D., Li L., Sun K. (2022). Charge reversal nano-systems for tumor therapy. J. Nanobiotechnol..

[106] Cao Z., Li D., Zhao L., Liu M., Ma P., Luo Y., Yang X. (2022). Bioorthogonal in situ assembly of nanomedicines as drug depots for extracellular drug delivery. Nat. Commun..

[107] Yu W., Liu R., Zhou Y., Gao H. (2020). Size-Tunable Strategies for a Tumor Targeted Drug Delivery System. ACS Cent. Sci..

[108] Ruan S., Hu C., Tang X., Cun X., Xiao W., Shi K., He Q., Gao H. (2016). Increased Gold Nanoparticle Retention in Brain Tumors by in Situ Enzyme-Induced Aggregation. ACS Nano.

[109] Hu Q., Sun W., Lu Y., Bomba H.N., Ye Y., Jiang T., Isaacson A.J., Gu Z. (2016). Tumor Microenvironment-Mediated Construction and Deconstruction of Extracellular Drug-Delivery Depots. Nano Lett..

[110] Malla R., Surepalli N., Farran B., Malhotra S.V., Nagaraju G.P. (2021). Reactive oxygen species (ROS): Critical roles in breast tumor microenvironment. Crit. Rev. Oncol. Hematol..

[111] Perillo B., Di Donato M., Pezone A., Di Zazzo E., Giovannelli P., Galasso G., Castoria G., Migliaccio A. (2020). ROS in cancer therapy: The bright side of the moon. Exp. Mol. Med..

[112] Tian H., Li W., Wang G., Tian Y., Yan J., Zhou S., Yu X., Li B., Dai Y. (2023). Self-Degradable Nanogels Reshape Immunosuppressive Tumor Microenvironment via Drug Repurposing Strategy to Reactivate Cytotoxic CD8^+^ T Cells. Adv. Sci..

[113] Zhou Y., Fan S., Feng L., Huang X., Chen X. (2021). Manipulating Intratumoral Fenton Chemistry for Enhanced Chemodynamic and Chemodynamic-Synergized Multimodal Therapy. Adv. Mater..

[114] Meng X., Zhang X., Liu M., Cai B., He N., Wang Z. (2020). Fenton reaction-based nanomedicine in cancer chemodynamic and synergistic therapy. Appl. Mater. Today.

[115] Liang J., Liu B. (2016). ROS-responsive drug delivery systems. Bioeng. Transl. Med..

[116] Saravanakumar G., Kim J., Kim W.J. (2017). Reactive-Oxygen-Species-Responsive Drug Delivery Systems: Promises and Challenges. Adv. Sci..

[117] Garcia-Mouronte E., Berna-Rico E., de Nicolas-Ruanes B., Azcarraga-Llobet C., Alonso-Martinez de Salinas L., Bea-Ardebol S. (2023). Imiquimod as Local Immunotherapy in the Management of Premalignant Cutaneous Conditions and Skin Cancer. Int. J. Mol. Sci..

[118] Bocanegra Gondan A.I., Ruiz-de-Angulo A., Zabaleta A., Gómez Blanco N., Cobaleda-Siles B.M., García-Granda M.J., Padro D., Llop J., Arnaiz B., Gato M. (2018). Effective cancer immunotherapy in mice by polyIC-imiquimod complexes and engineered magnetic nanoparticles. Biomaterials.

[119] Panaampon J., Zhou Y., Saengboonmee C. (2023). Metformin as a booster of cancer immunotherapy. Int. Immunopharmacol..

[120] Finisguerra V., Dvorakova T., Formenti M., Van Meerbeeck P., Mignion L., Gallez B., Van den Eynde B.J. (2023). Metformin improves cancer immunotherapy by directly rescuing tumor-infiltrating CD8 T lymphocytes from hypoxia-induced immunosuppression. J. Immunother. Cancer.

[121] Verdura S., Cuyàs E., Martin-Castillo B., Menendez J.A. (2019). Metformin as an archetype immuno-metabolic adjuvant for cancer immunotherapy. Oncoimmunology.

[122] Araújo Martins Y., Zeferino Pavan T., Fonseca Vianna Lopez R. (2021). Sonodynamic therapy: Ultrasound parameters and in vitro experimental configurations. Int. J. Pharm..

[123] Couture O., Foley J., Kassell N.F., Larrat B., Aubry J.-F. (2014). Review of ultrasound mediated drug delivery for cancer treatment: Updates from pre-clinical studies. Transl. Cancer Res..

[124] Tu L., Liao Z., Luo Z., Wu Y.-L., Herrmann A., Huo S. (2021). Ultrasound-controlled drug release and drug activation for cancer therapy. Exploration.

[125] Huang D., Wang J., Song C., Zhao Y. (2023). Ultrasound-responsive matters for biomedical applications. Innovation.

[126] Gao Y., Ma Q., Cao J., Shi Y., Wang J., Ma H., Sun Y., Song Y. (2021). Bifunctional alginate/chitosan stabilized perfluorohexane nanodroplets as smart vehicles for ultrasound and pH responsive delivery of anticancer agents. Int. J. Biol. Macromol..

[127] Zhang W., Shi Y., Abd Shukor S., Vijayakumaran A., Vlatakis S., Wright M., Thanou M. (2022). Phase-shift nanodroplets as an emerging sonoresponsive nanomaterial for imaging and drug delivery applications. Nanoscale.

[128] Mannaris C., Bau L., Grundy M., Gray M., Lea-Banks H., Seth A., Teo B., Carlisle R., Stride E., Coussios C.C. (2019). Microbubbles, Nanodroplets and Gas-Stabilizing Solid Particles for Ultrasound-Mediated Extravasation of Unencapsulated Drugs: An Exposure Parameter Optimization Study. Ultrasound Med. Biol..

[129] Lyu S.-Y., Kwon Y.-J., Joo H.-J., Park W.-B. (2004). Preparation of alginate/chitosan microcapsules and enteric coated granules of mistletoe lectin. Arch. Pharm. Res..

[130] Li J., Luo Y., Zeng Z., Cui D., Huang J., Xu C., Li L., Pu K., Zhang R. (2022). Precision cancer sono-immunotherapy using deep-tissue activatable semiconducting polymer immunomodulatory nanoparticles. Nat. Commun..

[131] Zhao P., Deng Y., Xiang G., Liu Y. (2021). Nanoparticle-Assisted Sonosensitizers and Their Biomedical Applications. Int. J. Nanomed..

[132] Son S., Kim J.H., Wang X., Zhang C., Yoon S.A., Shin J., Sharma A., Lee M.H., Cheng L., Wu J. (2020). Multifunctional sonosensitizers in sonodynamic cancer therapy. Chem. Soc. Rev..

[133] Rinaldi A., Caraffi R., Grazioli M.V., Oddone N., Giardino L., Tosi G., Vandelli M.A., Calzà L., Ruozi B., Duskey J.T. (2022). Applications of the ROS-Responsive Thioketal Linker for the Production of Smart Nanomedicines. Polymers.

[134] Ling X., Zhang S., Shao P., Wang P., Ma X., Bai M. (2015). Synthesis of a reactive oxygen species responsive heterobifunctional thioketal linker. Tetrahedron Lett..

[135] Chen W., Lian W., Yuan Y., Li M. (2019). The synergistic effects of oxaliplatin and piperlongumine on colorectal cancer are mediated by oxidative stress. Cell Death Dis..

[136] Jiang S., Xiao M., Sun W., Crespy D., Mailänder V., Peng X., Fan J., Landfester K. (2020). Synergistic Anticancer Therapy by Ovalbumin Encapsulation-Enabled Tandem Reactive Oxygen Species Generation. Angew. Chemie Int. Ed..

[137] Chen Y., Gao Y., Li Y., Wang K., Zhu J. (2019). Synergistic chemo-photodynamic therapy mediated by light-activated ROS-degradable nanocarriers. J. Mater. Chem. B.

[138] Lee K., Lee H.-J., Morino K., Sudo A., Endo T. (2011). Synthesis and photovoltaic behaviors of narrow-band-gap π-conjugated polymers composed of dialkoxybenzodithiophene- and thiophene-based fused aromatic rings. J. Polym. Sci. Part A Polym. Chem..

[139] Chang S.-W., Muto T., Kondo T., Liao M.-J., Horie M. (2017). Double acceptor donor–acceptor alternating conjugated polymers containing cyclopentadithiophene, benzothiadiazole and thienopyrroledione: Toward subtractive color organic photovoltaics. Polym. J..

[140] Qin R., Zhao C., Wang C.-J., Xu W., Zhao J.-Y., Lin Y., Yuan Y.-Y., Lin P.-C., Li Y., Zhao S. (2021). Tryptophan potentiates CD8^+^ T cells against cancer cells by TRIP12 tryptophanylation and surface PD-1 downregulation. J. Immunother. Cancer.

[141] Sun J.-J., Chen Y.-C., Huang Y.-X., Zhao W.-C., Liu Y.-H., Venkataramanan R., Lu B.-F., Li S. (2017). Programmable co-delivery of the immune checkpoint inhibitor NLG919 and chemotherapeutic doxorubicin via a redox-responsive immunostimulatory polymeric prodrug carrier. Acta Pharmacol. Sin..

[142] Akinleye A., Rasool Z. (2019). Immune checkpoint inhibitors of PD-L1 as cancer therapeutics. J. Hematol. Oncol..

[143] Twomey J.D., Zhang B. (2021). Cancer Immunotherapy Update: FDA-Approved Checkpoint Inhibitors and Companion Diagnostics. AAPS J..

[144] Ashar H., Ranjan A. (2023). Immunomodulation and targeted drug delivery with high intensity focused ultrasound (HIFU): Principles and mechanisms. Pharmacol. Ther..

[145] van den Bijgaart R.J.E., Eikelenboom D.C., Hoogenboom M., Fütterer J.J., den Brok M.H., Adema G.J. (2017). Thermal and mechanical high-intensity focused ultrasound: Perspectives on tumor ablation, immune effects and combination strategies. Cancer Immunol. Immunother..

[146] Zhou Y.-F. (2011). High intensity focused ultrasound in clinical tumor ablation. World J. Clin. Oncol..

[147] Wu D., Jin X., Wang X., Ma B., Lou C., Qu H., Zheng J., Zhang B., Yan X., Wang Y. (2019). Engineering temperature-sensitive plateletsomes as a tailored chemotherapy platform in combination with HIFU ablation for cancer treatment. Theranostics.

[148] Yap M.L., McFadyen J.D., Wang X., Ziegler M., Chen Y.-C., Willcox A., Nowell C.J., Scott A.M., Sloan E.K., Hogarth P.M. (2019). Activated platelets in the tumor microenvironment for targeting of antibody-drug conjugates to tumors and metastases. Theranostics.

[149] Morris K., Schnoor B., Papa A.-L. (2022). Platelet cancer cell interplay as a new therapeutic target. Biochim. Biophys. Acta. Rev. Cancer.

[150] Hu Q., Sun W., Qian C., Wang C., Bomba H.N., Gu Z. (2015). Anticancer Platelet-Mimicking Nanovehicles. Adv. Mater..

[151] Elaskalani O., Berndt M.C., Falasca M., Metharom P. (2017). Targeting Platelets for the Treatment of Cancer. Cancers.

[152] Xu L., Gao F., Fan F., Yang L. (2018). Platelet membrane coating coupled with solar irradiation endows a photodynamic nanosystem with both improved antitumor efficacy and undetectable skin damage. Biomaterials.

[153] Tan Y.-N., Li Y.-P., Huang J.-D., Luo M., Li S.-S., Lee A.W.-M., Hu F.-Q., Guan X.-Y. (2021). Thermal-sensitive lipid nanoparticles potentiate anti-PD therapy through enhancing drug penetration and T lymphocytes infiltration in metastatic tumor. Cancer Lett..

[154] Su J., Sun H., Meng Q., Yin Q., Zhang P., Zhang Z., Yu H., Li Y. (2016). Bioinspired Nanoparticles with NIR-Controlled Drug Release for Synergetic Chemophotothermal Therapy of Metastatic Breast Cancer. Adv. Funct. Mater..

[155] Kang Y., Kim J., Park J., Lee Y.M., Saravanakumar G., Park K.M., Choi W., Kim K., Lee E., Kim C. (2019). Tumor vasodilation by N-Heterocyclic carbene-based nitric oxide delivery triggered by high-intensity focused ultrasound and enhanced drug homing to tumor sites for anti-cancer therapy. Biomaterials.

[156] Yasuda H. (2008). Solid tumor physiology and hypoxia-induced chemo/radio-resistance: Novel strategy for cancer therapy: Nitric oxide donor as a therapeutic enhancer. Nitric Oxide Biol. Chem..

[157] Kim J., Saravanakumar G., Choi H.W., Park D., Kim W.J. (2014). A platform for nitric oxide delivery. J. Mater. Chem. B.

[158] Carpenter A.W., Schoenfisch M.H. (2012). Nitric oxide release: Part II. Therapeutic applications. Chem. Soc. Rev..

[159] Studenovsky M., Sivak L., Sedlacek O., Konefal R., Horkova V., Etrych T., Kovar M., Rihova B., Sirova M. (2018). Polymer nitric oxide donors potentiate the treatment of experimental solid tumours by increasing drug accumulation in the tumour tissue. J. Control. Release.

[160] Deepagan V.G., Ko H., Kwon S., Rao N.V., Kim S.K., Um W., Lee S., Min J., Lee J., Choi K.Y. (2018). Intracellularly Activatable Nanovasodilators To Enhance Passive Cancer Targeting Regime. Nano Lett..

[161] Tahara Y., Yoshikawa T., Sato H., Mori Y., Zahangir M.H., Kishimura A., Mori T., Katayama Y. (2017). Encapsulation of a nitric oxide donor into a liposome to boost the enhanced permeation and retention (EPR) effect. Medchemcomm.

[162] Park J., Song H., Kim Y., Eun B., Kim Y., Bae D.Y., Park S., Rhee Y.M., Kim W.J., Kim K. (2015). N-Heterocyclic Carbene Nitric Oxide Radicals. J. Am. Chem. Soc..

[163] Shamsipur M., Ghavidast A., Pashabadi A. (2023). Phototriggered structures: Latest advances in biomedical applications. Acta Pharm. Sin. B.

[164] Lee H.P., Gaharwar A.K. (2020). Light-Responsive Inorganic Biomaterials for Biomedical Applications. Adv. Sci..

[165] Yun S.H., Kwok S.J.J. (2017). Light in diagnosis, therapy and surgery. Nat. Biomed. Eng..

[166] Jaque D., Martínez Maestro L., del Rosal B., Haro-Gonzalez P., Benayas A., Plaza J.L., Martín Rodríguez E., García Solé J. (2014). Nanoparticles for photothermal therapies. Nanoscale.

[167] Wu S., Butt H.J. (2017). Near-infrared photochemistry at interfaces based on upconverting nanoparticles. Phys. Chem. Chem. Phys..

[168] Padalkar M.V., Pleshko N. (2015). Wavelength-dependent penetration depth of near infrared radiation into cartilage. Analyst.

[169] Jalani G., Tam V., Vetrone F., Cerruti M. (2018). Seeing, Targeting and Delivering with Upconverting Nanoparticles. J. Am. Chem. Soc..

[170] Chen G., Qiu H., Prasad P.N., Chen X. (2014). Upconversion Nanoparticles: Design, Nanochemistry, and Applications in Theranostics. Chem. Rev..

[171] Escudero A., Carrillo-Carrión C., Castillejos M.C., Romero-Ben E., Rosales-Barrios C., Khiar N. (2021). Photodynamic therapy: Photosensitizers and nanostructures. Mater. Chem. Front..

[172] Abrahamse H., Hamblin M.R. (2016). New photosensitizers for photodynamic therapy. Biochem. J..

[173] Baskaran R., Lee J., Yang S.-G. (2018). Clinical development of photodynamic agents and therapeutic applications. Biomater. Res..

[174] Han H.S., Choi K.Y. (2021). Advances in Nanomaterial-Mediated Photothermal Cancer Therapies: Toward Clinical Applications. Biomedicines.

[175] Li C., Cheng Y., Li D., An Q., Zhang W., Zhang Y., Fu Y. (2022). Antitumor Applications of Photothermal Agents and Photothermal Synergistic Therapies. Int. J. Mol. Sci..

[176] Zhao L., Zhang X., Wang X., Guan X., Zhang W., Ma J. (2021). Recent advances in selective photothermal therapy of tumor. J. Nanobiotechnol..

[177] Islam M.R., Irvine J., Serpe M.J. (2015). Photothermally Induced Optical Property Changes of Poly(N-isopropylacrylamide) Microgel-Based Etalons. ACS Appl. Mater. Interfaces.

[178] Zhang C., Jing X., Guo L., Cui C., Hou X., Zuo T., Liu J., Shi J., Liu X., Zuo X. (2021). Remote Photothermal Control of DNA Origami Assembly in Cellular Environments. Nano Lett..

[179] Poon L., Zandberg W., Hsiao D., Erno Z., Sen D., Gates B.D., Branda N.R. (2010). Photothermal Release of Single-Stranded DNA from the Surface of Gold Nanoparticles Through Controlled Denaturating and Au−S Bond Breaking. ACS Nano.

[180] Bao J., Tu H., Li J., Li Y., Yu S., Gao J., Lei K., Zhang F., Li J. (2022). Applications of phase change materials in smart drug delivery for cancer treatment. Front. Bioeng. Biotechnol..

[181] Lee J., Park H., Kim W.J. (2015). Nano “Chocolate Waffle” for near-IR Responsive Drug Releasing System. Small.

[182] Bakhtiari A.B.S., Hsiao D., Jin G., Gates B.D., Branda N.R. (2009). An efficient method based on the photothermal effect for the release of molecules from metal nanoparticle surfaces. Angew. Chem. Int. Ed. Engl..

[183] Jiang Y., Huang J., Xu C., Pu K. (2021). Activatable polymer nanoagonist for second near-infrared photothermal immunotherapy of cancer. Nat. Commun..

[184] Zhang L., Chu C., Lin X., Sun R., Li Z., Chen S., Liu Y., Wu J., Yu Z., Liu X. (2023). Tunable Nanoparticles with Aggregation-Induced Emission Heater for Precise Synergistic Photothermal and Thermodynamic Oral Cancer Therapy of Patient-Derived Tumor Xenograft. Adv. Sci..

[185] Geng S., Guo M., Zhan G., Shi D., Shi L., Gan L., Zhao Y., Yang X. (2023). NIR-triggered ligand-presenting nanocarriers for enhancing synergistic photothermal-chemotherapy. J. Control. Release.

[186] Pu X.-Q., Ju X.-J., Zhang L., Cai Q.-W., Liu Y.-Q., Peng H.-Y., Xie R., Wang W., Liu Z., Chu L.-Y. (2021). Novel Multifunctional Stimuli-Responsive Nanoparticles for Synergetic Chemo–Photothermal Therapy of Tumors. ACS Appl. Mater. Interfaces.

[187] Yang L., Fan X., Zhang J., Ju J. (2020). Preparation and Characterization of Thermoresponsive Poly(N-Isopropylacrylamide) for Cell Culture Applications. Polymers.

[188] Nagase K., Yamato M., Kanazawa H., Okano T. (2018). Poly(N-isopropylacrylamide)-based thermoresponsive surfaces provide new types of biomedical applications. Biomaterials.

[189] Huang D., Zhou Y., Xiang Y., Shu M., Chen H., Yang B., Liao X. (2018). Polyurethane/doxorubicin nanoparticles based on electrostatic interactions as pH-sensitive drug delivery carriers. Polym. Int..

[190] López-Muñoz R., Treviño M.E., Castellanos F., Morales G., Rodríguez-Fernández O., Saavedra S., Licea-Claverie A., Saade H., Enríquez-Medrano F.J., López R.G. (2021). Loading of doxorubicin on poly(methyl methacrylate-co-methacrylic acid) nanoparticles and release study. J. Biomater. Sci. Polym. Ed..

[191] Geisow M.J., Evans W.H. (1984). pH in the endosome: Measurements during pinocytosis and receptor-mediated endocytosis. Exp. Cell Res..

[192] Wallabregue A., Moreau D., Sherin P., Moneva Lorente P., Jarolímová Z., Bakker E., Vauthey E., Gruenberg J., Lacour J. (2016). Selective Imaging of Late Endosomes with a pH-Sensitive Diazaoxatriangulene Fluorescent Probe. J. Am. Chem. Soc..

[193] Fan S., Lin W., Huang Y., Xia J., Xu J.-F., Zhang J., Pi J. (2022). Advances and Potentials of Polydopamine Nanosystem in Photothermal-Based Antibacterial Infection Therapies. Front. Pharmacol..

[194] Park J., Moon H., Hong S. (2019). Recent advances in melanin-like nanomaterials in biomedical applications: A mini review. Biomater. Res..

[195] Liu Y., Ai K., Liu J., Deng M., He Y., Lu L. (2013). Dopamine-Melanin Colloidal Nanospheres: An Efficient Near-Infrared Photothermal Therapeutic Agent for In Vivo Cancer Therapy. Adv. Mater..

[196] Fernández M., Javaid F., Chudasama V. (2018). Advances in targeting the folate receptor in the treatment/imaging of cancers. Chem. Sci..

[197] Zwicke G.L., Ali Mansoori G., Jeffery C.J. (2012). Utilizing the folate receptor for active targeting of cancer nanotherapeutics. Nano Rev..

[198] Ghosh D., Hossain M., Saha C., Dey S.K., Kumar G.S. (2012). Intercalation and induction of strand breaks by adriamycin and daunomycin: A study with human genomic DNA. DNA Cell Biol..

[199] Hendry L.B., Mahesh V.B., Bransome E.D.J., Ewing D.E. (2007). Small molecule intercalation with double stranded DNA: Implications for normal gene regulation and for predicting the biological efficacy and genotoxicity of drugs and other chemicals. Mutat. Res..

[200] Mukherjee A., Lavery R., Bagchi B., Hynes J.T. (2008). On the Molecular Mechanism of Drug Intercalation into DNA: A Simulation Study of the Intercalation Pathway, Free Energy, and DNA Structural Changes. J. Am. Chem. Soc..

[201] Xiong X., Wu C., Zhou C., Zhu G., Chen Z., Tan W. (2013). Responsive DNA-based hydrogels and their applications. Macromol. Rapid Commun..

[202] Singh A., Maity A., Singh N. (2022). Structure and Dynamics of dsDNA in Cell-like Environments. Entropy.

[203] Yang Y., Cai X., Shi M., Zhang X., Pan Y., Zhang Y., Ju H., Cao P. (2023). Biomimetic retractable DNA nanocarrier with sensitive responsivity for efficient drug delivery and enhanced photothermal therapy. J. Nanobiotechnol..

[204] Tian H., Zhang J., Zhang H., Jiang Y., Song A., Luan Y. (2020). Low side-effect and heat-shock protein-inhibited chemo-phototherapy nanoplatform via co-assembling strategy of biotin-tailored IR780 and quercetin. Chem. Eng. J..

[205] Lee S.H., Lee E.J., Min K.H., Hur G.Y., Lee S.H., Lee S.Y., Kim J.H., Shin C., Shim J.J., In K.H. (2015). Quercetin Enhances Chemosensitivity to Gemcitabine in Lung Cancer Cells by Inhibiting Heat Shock Protein 70 Expression. Clin. Lung Cancer.

[206] Dolmans D.E.J.G.J., Fukumura D., Jain R.K. (2003). Photodynamic therapy for cancer. Nat. Rev. Cancer.

[207] Žárská L., Malá Z., Langová K., Malina L., Binder S., Bajgar R., Henke P., Mosinger J., Kolářová H. (2022). Biological Evaluation of Photodynamic Effect Mediated by Nanoparticles with Embedded Porphyrin Photosensitizer. Int. J. Mol. Sci..

[208] Zhou Z., Song J., Nie L., Chen X. (2016). Reactive oxygen species generating systems meeting challenges of photodynamic cancer therapy. Chem. Soc. Rev..

[209] Castano A.P., Demidova T.N., Hamblin M.R. (2004). Mechanisms in photodynamic therapy: Part one-photosensitizers, photochemistry and cellular localization. Photodiagn. Photodyn. Ther..

[210] Saravanakumar G., Park H., Kim J., Park D., Lim J., Lee J., Kim W.J. (2020). Polymersomes with singlet oxygen-labile poly(β-aminoacrylate) membrane for NIR light-controlled combined chemo-phototherapy. J. Control. Release.

[211] Saravanakumar G., Park H., Kim J., Park D., Pramanick S., Kim D.H., Kim W.J. (2018). Miktoarm Amphiphilic Block Copolymer with Singlet Oxygen-Labile Stereospecific β-Aminoacrylate Junction: Synthesis, Self-Assembly, and Photodynamically Triggered Drug Release. Biomacromolecules.

[212] He B., Su H., Bai T., Wu Y., Li S., Gao M., Hu R., Zhao Z., Qin A., Ling J. (2017). Spontaneous Amino-yne Click Polymerization: A Powerful Tool toward Regio- and Stereospecific Poly(β-aminoacrylate)s. J. Am. Chem. Soc..

[213] Zhang L., Jin D., Stenzel M.H. (2021). Polymer-Functionalized Upconversion Nanoparticles for Light/Imaging-Guided Drug Delivery. Biomacromolecules.

[214] Ovais M., Mukherjee S., Pramanik A., Das D., Mukherjee A., Raza A., Chen C. (2020). Designing Stimuli-Responsive Upconversion Nanoparticles that Exploit the Tumor Microenvironment. Adv. Mater..

[215] LeValley P.J., Neelarapu R., Sutherland B.P., Dasgupta S., Kloxin C.J., Kloxin A.M. (2020). Photolabile Linkers: Exploiting Labile Bond Chemistry to Control Mode and Rate of Hydrogel Degradation and Protein Release. J. Am. Chem. Soc..

[216] Yang Y., Velmurugan B., Liu X., Xing B. (2013). NIR Photoresponsive Crosslinked Upconverting Nanocarriers Toward Selective Intracellular Drug Release. Small.

[217] Leriche G., Chisholm L., Wagner A. (2012). Cleavable linkers in chemical biology. Bioorg. Med. Chem..

[218] Liu Z., Feng Z., Chen M., Zhan J., Wu R., Shi Y., Xue Y., Liu R., Zhu J.-J., Zhang J. (2023). An orthogonally activatable CRISPR-Cas13d nanoprodrug to reverse chemoresistance for enhanced chemo-photodynamic therapy. Chem. Sci..

[219] Materón E.M., Miyazaki C.M., Carr O., Joshi N., Picciani P.H.S., Dalmaschio C.J., Davis F., Shimizu F.M. (2021). Magnetic nanoparticles in biomedical applications: A review. Appl. Surf. Sci. Adv..

[220] Fatima H., Charinpanitkul T., Kim K.-S. (2021). Fundamentals to Apply Magnetic Nanoparticles for Hyperthermia Therapy. Nanomaterials.

[221] Liu X., Zhang Y., Wang Y., Zhu W., Li G., Ma X., Zhang Y., Chen S., Tiwari S., Shi K. (2020). Comprehensive understanding of magnetic hyperthermia for improving antitumor therapeutic efficacy. Theranostics.

[222] Chandrasekharan P., Tay Z.W., Hensley D., Zhou X.Y., Fung B.K.L., Colson C., Lu Y., Fellows B.D., Huynh Q., Saayujya C. (2020). Using magnetic particle imaging systems to localize and guide magnetic hyperthermia treatment: Tracers, hardware, and future medical applications. Theranostics.

[223] Jiang M., Liu Q., Zhang Y., Wang H., Zhang J., Chen M., Yue Z., Wang Z., Wei X., Shi S. (2022). Construction of magnetic drug delivery system and its potential application in tumor theranostics. Biomed. Pharmacother..

[224] Price P.M., Mahmoud W.E., Al-Ghamdi A.A., Bronstein L.M. (2018). Magnetic Drug Delivery: Where the Field Is Going. Front. Chem..

[225] Liu J.F., Jang B., Issadore D., Tsourkas A. (2019). Use of magnetic fields and nanoparticles to trigger drug release and improve tumor targeting. WIREs Nanomed. Nanobiotechnol..

[226] Dwivedi P., Kiran S., Han S., Dwivedi M., Khatik R., Fan R., Mangrio F.A., Du K., Zhu Z., Yang C. (2020). Magnetic Targeting and Ultrasound Activation of Liposome–Microbubble Conjugate for Enhanced Delivery of Anticancer Therapies. ACS Appl. Mater. Interfaces.

[227] Rivera-Rodriguez A., Rinaldi-Ramos C.M. (2021). Emerging Biomedical Applications Based on the Response of Magnetic Nanoparticles to Time-Varying Magnetic Fields. Annu. Rev. Chem. Biomol. Eng..

[228] Gao M., Feng K., Zhang X., Ruan Y., Zhao G., Liu H., Sun X. (2023). Nanoassembly with self-regulated magnetic thermal therapy and controlled immuno-modulating agent release for improved immune response. J. Control. Release.

[229] Zhang Z., Zhang Q., Xie J., Zhong Z., Deng C. (2021). Enzyme-responsive micellar JQ1 induces enhanced BET protein inhibition and immunotherapy of malignant tumors. Biomater. Sci..

[230] Wang H., Liu G., Jin X., Song S., Chen S., Zhou P., Li H., Liang J., Li B., Zhang C. (2022). BET inhibitor JQ1 enhances anti-tumor immunity and synergizes with PD-1 blockade in CRC. J. Cancer.

[231] Sauvage D., Bosseler M., Viry E., Kanli G., Oudin A., Berchem G., Keunen O., Janji B. (2022). The BET Protein Inhibitor JQ1 Decreases Hypoxia and Improves the Therapeutic Benefit of Anti-PD-1 in a High-Risk Neuroblastoma Mouse Model. Cells.

[232] Lei L., Xie X., He L., Chen K., Lv Z., Zhou B., Li Y., Hu W., Zhou Z. (2021). The bromodomain and extra-terminal domain inhibitor JQ1 synergistically sensitizes human colorectal cancer cells to topoisomerase I inhibitors through repression of Mre11-mediated DNA repair pathway. Investig. New Drugs.

[233] Liu Y., Wang Z., Zhang H., Lang L., Ma Y., He Q., Lu N., Huang P., Liu Y., Song J. (2016). A photothermally responsive nanoprobe for bioimaging based on Edman degradation. Nanoscale.

[234] Tang L., Yang Z., Zhou Z., Ma Y., Kiesewetter D.O., Wang Z., Fan W., Zhu S., Zhang M., Tian R. (2019). A Logic-Gated Modular Nanovesicle Enables Programmable Drug Release for On-Demand Chemotherapy. Theranostics.

[235] Milani V., Noessner E. (2006). Effects of thermal stress on tumor antigenicity and recognition by immune effector cells. Cancer Immunol. Immunother..

[236] Evans S.S., Repasky E.A., Fisher D.T. (2015). Fever and the thermal regulation of immunity: The immune system feels the heat. Nat. Rev. Immunol..

[237] Rana A., Adhikary M., Singh P.K., Das B.C., Bhatnagar S. (2023). “Smart” drug delivery: A window to future of translational medicine. Front. Chem..

[238] Sameiyan E., Bagheri E., Dehghani S., Ramezani M. (2021). Acta Biomaterialia Aptamer-based ATP-responsive delivery systems for cancer diagnosis and treatment. Acta Biomater..

[239] Mo R., Jiang T., DiSanto R., Tai W., Gu Z. (2014). ATP-triggered anticancer drug delivery. Nat. Commun..

[240] Mo R., Jiang T., Sun W., Gu Z. (2015). ATP-responsive DNA-graphene hybrid nanoaggregates for anticancer drug delivery. Biomaterials.

[241] Zhao M., Song X., Lu J., Liu S., Sha X., Wang Q., Cao X., Xu K., Li J. (2022). DNA aptamer-based dual-responsive nanoplatform for targeted MRI and combination therapy for cancer. RSC Adv..

[242] Yang Y., Liu J., Sun X., Feng L., Zhu W., Liu Z., Chen M. (2016). Near-infrared light-activated cancer cell targeting and drug delivery with aptamer-modified nanostructures. Nano Res..

[243] Zhang Y., Hou Z., Ge Y., Deng K., Liu B., Li X., Li Q., Cheng Z., Ma P., Li C. (2015). DNA-Hybrid-Gated Photothermal Mesoporous Silica Nanoparticles for NIR-Responsive and Aptamer-Targeted Drug Delivery. ACS Appl. Mater. Interfaces.

[244] Cai S., Yan J., Xiong H., Wu Q., Xing H., Liu Y. (2020). Aptamer-functionalized molybdenum disulfide nanosheets for tumor cell targeting and lysosomal acidic environment/NIR laser responsive drug delivery to realize synergetic chemo-photothermal therapeutic effects. Int. J. Pharm..

[245] Ambekar R.S., Kandasubramanian B. (2019). A polydopamine-based platform for anti-cancer drug delivery. Biomater. Sci..

[246] Dai L., Wei D., Zhang J., Shen T., Zhao Y., Liang J., Ma W., Zhang L., Liu Q., Zheng Y. (2021). Aptamer-conjugated mesoporous polydopamine for docetaxel targeted delivery and synergistic photothermal therapy of prostate cancer. Cell Prolif..

[247] Yang L., Sun H., Liu Y., Hou W., Yang Y., Cai R., Cui C., Zhang P., Pan X., Li X. (2018). Self-Assembled Aptamer-Grafted Hyperbranched Polymer Nanocarrier for Targeted and Photoresponsive Drug Delivery. Angew. Chem..

[248] Chen Z., Peng Y., Li Y., Xie X., Wei X., Yang G., Zhang H., Li N., Li T., Qin X. (2021). Aptamer-Dendrimer Functionalized Magnetic Nano-Octahedrons: Theranostic Drug/Gene Delivery Platform for Near-Infrared/Magnetic Resonance Imaging-Guided Magnetochemotherapy. ACS Nano.

[249] Ni S., Zhuo Z., Pan Y., Yu Y., Li F., Liu J., Wang L., Wu X., Li D., Wan Y. (2021). Recent Progress in Aptamer Discoveries and Modifications for Therapeutic Applications. ACS Appl. Mater. Interfaces.

[250] Chandola C., Neerathilingam M., Tyagi R.K., Garg N., Shukla R., Bisen P.S. (2019). Aptamers for Targeted Delivery: Current Challenges and Future Opportunities.

[251] Lakhin A.V., Tarantul V.Z., Gening L. (2013). V Aptamers: Problems, solutions and prospects. Acta Naturae.

[252] Gao F., Yin J., Chen Y., Guo C., Hu H., Su J. (2022). Recent advances in aptamer-based targeted drug delivery systems for cancer therapy. Front. Bioeng. Biotechnol..

[253] Venkatesan S., Chanda K., Balamurali M.M. (2023). Recent Advancements of Aptamers in Cancer Therapy. ACS Omega.

[254] Khabbazian M., Jabbari H. (2022). AI-powered aptamer generation. Nat. Comput. Sci..

